# Macrophage‐derived extracellular vesicles regulate follicular activation and improve ovarian function in old mice by modulating local environment

**DOI:** 10.1002/ctm2.1071

**Published:** 2022-10-13

**Authors:** Yue Xiao, Xiaoxu Peng, Yue Peng, Chi Zhang, Wei Liu, Weijie Yang, Xiaowei Dou, Yuying Jiang, Yaxuan Wang, Shuo Yang, Wenpei Xiang, Tinghe Wu, Jing Li

**Affiliations:** ^1^ State Key Laboratory of Reproductive Medicine Nanjing Medical University Nanjing Jiangsu China; ^2^ Women's Hospital School of Medicine Zhejiang University Zhejiang Hangzhou China; ^3^ Bayer Healthcare Company Limited Pudong Shanghai China; ^4^ Assisted Reproduction Unit, Department of Obstetrics and Gynecology, Sir Run Shaw Hospital Zhejiang University School of Medicine, Key Laboratory of Reproductive Dysfunction Management of Zhejiang Province Zhejiang Hangzhou China; ^5^ Department of Obstetrics and Gynecology The Second Affiliated Hospital of Nanjing Medical University Nanjing Jiangsu China; ^6^ Department of Immunology, Key Laboratory of Immunological Environment and Disease, Gusu School, State Key Laboratory of Reproductive Medicine, Jiangsu Key Lab of Cancer Biomarkers, Prevention and Treatment, Collaborative Innovation Center for Personalized Cancer Medicine, Center for Global Health Nanjing Medical University Nanjing Jiangsu China; ^7^ Family Planning Research Institute/Center of Reproductive Medicine Tongji Medical College Huazhong University of Science and Technology Wuhan Hubei China; ^8^ State Key Laboratory of Translational Medicine and Innovative Drug Development Jiangsu Simcere Pharmaceutical Co., Ltd. Nanjing Jiangsu China

**Keywords:** extracellular vesicles, macrophage, ovarian ageing, primordial follicle

## Abstract

In mammals, ovarian function is dependent on the primordial follicle pool and the rate of primordial follicle activation determines a female's reproductive lifespan. Ovarian ageing is characterised by chronic low‐grade inflammation with accelerated depletion of primordial follicles and deterioration of oocyte quality. Macrophages (Mφs) play critical roles in multiple aspects of ovarian functions; however, it remains unclear whether Mφs modulate the primordial follicle pool and what is their role in ovarian ageing. Here, by using super‐ or naturally ovulated mouse models, we demonstrated for the first time that ovulation‐induced local inflammation acted as the driver for selective activation of surrounding primordial follicles in each estrous cycle. This finding was related to infiltrating Mφs in ovulatory follicles and the dynamic changes of the two polarised Mφs, M1 and M2 Mφs, during the process. Further studies on newborn ovaries cocultured with different subtypes of Mφs demonstrated the stimulatory effect of M1 Mφs on primordial follicles, whereas M2 Mφs maintained follicles in a dormant state. The underlying mechanism was associated with the differential regulation of the Phosphatidylinositol 3‐kinase/Mechanistic target of rapamycin (PI3K/mTOR) signaling pathway through secreted extracellular vesicles (EVs) and the containing specific miRNAs miR‐107 (M1 Mφs) and miR‐99a‐5p (M2 Mφs). In aged mice, the intravenous injection of M2‐EVs improved ovarian function and ameliorated the inflammatory microenvironment within the ovary. Thus, based on the anti‐ageing effects of M2 Mφs in old mice, M2‐EVs may represent a new approach to improve inflammation‐related infertility in women.

## Graphical abstract

- Ovulation induced local inflammation environment selectively activates primordial follicles.
- M1 Mφs have a stimulatory effect on primordial follicles while M2 Mφs maintain them in a dormant state.
- M2‐EVs in vivo treatment ameliorate the inflammatory microenvironment within the ovary and improve ovarian function in aged mice.

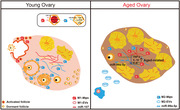

## 1 INTRODUCTION

In recent years, with changes in concepts of fertility and social factors, the age at which women have their first child has increased. This change has created a major health and societal challenge because advanced maternal age is related to the rise in adverse maternal and foetal outcomes.^1^, ^2^ Females are born with a finite number of primordial follicles. These follicles are preserved in a dormant state and are consumed progressively with age.^3^ Although female fertility peaks at approximately 25 years, surprisingly, the decrease in female fecundity starts in the 30s. During this period, the number of follicles drops from 130 000 at 25 years of age to only 30 000 at the age of 35 years, and follicle depletion occurs at menopause (∼51 years of age), when fewer than 1000 follicles remain.^4^ Until now, the mechanism of this rapid decline in ovarian reserve has been poorly understood. However, it has been generally accepted that an altered microenvironment in the ovary contributes to consumption of the ovarian reserve and declines in oocyte quality.

The activation of primordial follicles is not random; rather, it requires the coordinated efforts of many growth factors and cytokines with impacts in an autocrine or paracrine manner. In the past few decades, the use of transgenic mouse models has revealed that the intraoocyte PI3K/mTOR pathways are essential for the transition from primordial to primary follicles.^5^, ^6^ Oocyte‐specific knockout of inhibitors of these pathways, such as Forkhead box O3 (FOXO3a), phosphatase and tensin homolog deleted on chromosome ten (PTEN), ribosome protein S6 (RPS6) and tuberous sclerosis 1/tuberous sclerosis 2 (TSC1/TSC2), led to global activation of all primordial follicles, which inevitably caused depletion of the primordial follicle pool and premature ovarian failure (POF).^7^, ^8^, ^9^ Further study revealed the essential role of the pregranulosa mTOR signalling pathway in transmitting surrounding microenvironmental signals to activate primordial oocytes through the kit ligand/c‐kitproto‐oncogeneprotein‐phosphatidylinositol 3‐kinase (KITL/KIT‐PI3K) signaling pathway. Under physiological conditions, only a limited number of primordial follicles (approximately 1000) are recruited into the growth state in each follicle wave.^10^ Until now, it was unclear how selective follicular activation occurs at any given time. In our previous research, we established a partial ovarian resection murine model and found that the activation of primordial follicles was limited near the trauma, where the altered microenvironment contributed to the process by sequential activation of the mTOR signalling pathway from stroma to oocyte.^11^ Our results also revealed a significant increase in inflammation in injured ovaries. It has been reported that neonatal lipopolysaccharide (LPS) treatment immediately interferes with the delicate process of primordial follicle pool assembly and the subsequent activation of follicular development by stimulating an acute pro‐inflammatory response.^12^, ^13^ Additionally, chemo‐ or radiotherapy‐induced depletion of the primordial follicle pool was related to the activation of inflammatory signalling pathways.^14^ In females, ovulation has been linked to an inflammatory response, which is initiated by the luteinising hormone (LH) surge and is accompanied by an influx of leucocytes in the preovulatory ovary. The sequential events during ovulation are spatially restricted to the specific inflammatory microenvironment within the follicle or surrounding interstitial compartments, allowing successful expulsion of the cumulus–oocyte complex from the ruptured follicle.^15^ Thus, there is the possibility that the inflammatory microenvironment surrounding ovulating follicles initiates the localised activation of primordial follicles.

Macrophages (Mφs) are the most abundant leucocytes in ovaries. Consistent with their characteristics in other organs, Mφs in the ovary display high levels of heterogeneity and plasticity in their functional responses to different stimuli.^16^ Traditionally, Mφs can be activated and polarised to M1 and M2 Mφs according to their exposure to pro‐ or anti‐inflammatory signals. M1 Mφs mainly function in eliminating intracellular pathogens, whereas M2 Mφs participate in tissue remodelling and repair, as well as the resolution of inflammation.^17^ It is now clear that Mφs play important roles in folliculogenesis, ovulation and luteolysis.^18^ The balance between Mφ phenotypic states dictates the immunological milieu within the ovary, and many ovarian pathological conditions, such as ovarian ageing, polycystic ovarian syndrome (PCOS) and ovarian cancer, have been linked to abnormal Mφ activity.^19^ However, there has been no evidence indicating a direct interaction between Mφs and primordial follicles. The participation of Mφs in follicle growth has been suggested by genetic mouse models, and different Mφ populations appear to have distinct impacts on this process. The depletion of classically activated M1‐like Mφs (CD11c^+^) causes follicular impairment and haemorrhaging, whereas the depletion of alternatively activated M2‐like Mφs (CD206^+^) does not.^20^ Mice deficient in transforming growth factor β1 (TGF‐β1), a key factor that promotes Mφs alternate activation (M2), exhibit impaired progesterone (P4) synthesis in early pregnancy. A previous study revealed dynamic changes in Mφs during the ovarian cycle. As the major producers of various cytokines and chemokines, Mφs infiltrate preovulatory follicles when ovulation is initiated. In addition to their role in the recruitment of leucocytes, Mφs have been shown to directly participate in extracellular matrix (ECM) breakdown and tissue remodelling upon ovulation through the activities of matrix metalloproteinases (MMPs).^21^ In the ageing ovary, an age‐associated shift in Mφ activation status implies involvement in the increase in basal inflammation.^22^, ^23^ If ovarian inflammation is related to the activation of primordial follicles, what is the role of Mφs in this process, and how do these cells regulate follicular activation?

Herein, our study revealed for the first time the relationship between ovulation and selective primordial follicle activation. Ovulation‐induced local Mφ infiltration participated in the activation of the surrounding primordial follicles. In the in vitro coculture model of newborn ovaries, M1 Mφs were found to activate follicles, whereas M2 Mφs maintained primordial follicles in a dormant state. We then explored the differential regulation of primordial follicle activation and ovarian function by Mφ‐derived extracellular vesicles (Mφ‐EVs) in aged mice. Our results revealed a new mechanism of selective follicular activation and demonstrated that Mφ‐EVs can regulate the immune microenvironment in aged ovaries.

## 2 RESULTS

### 2.1 Ovulation stimulates the activation of primordial follicles

**FIGURE 1:**
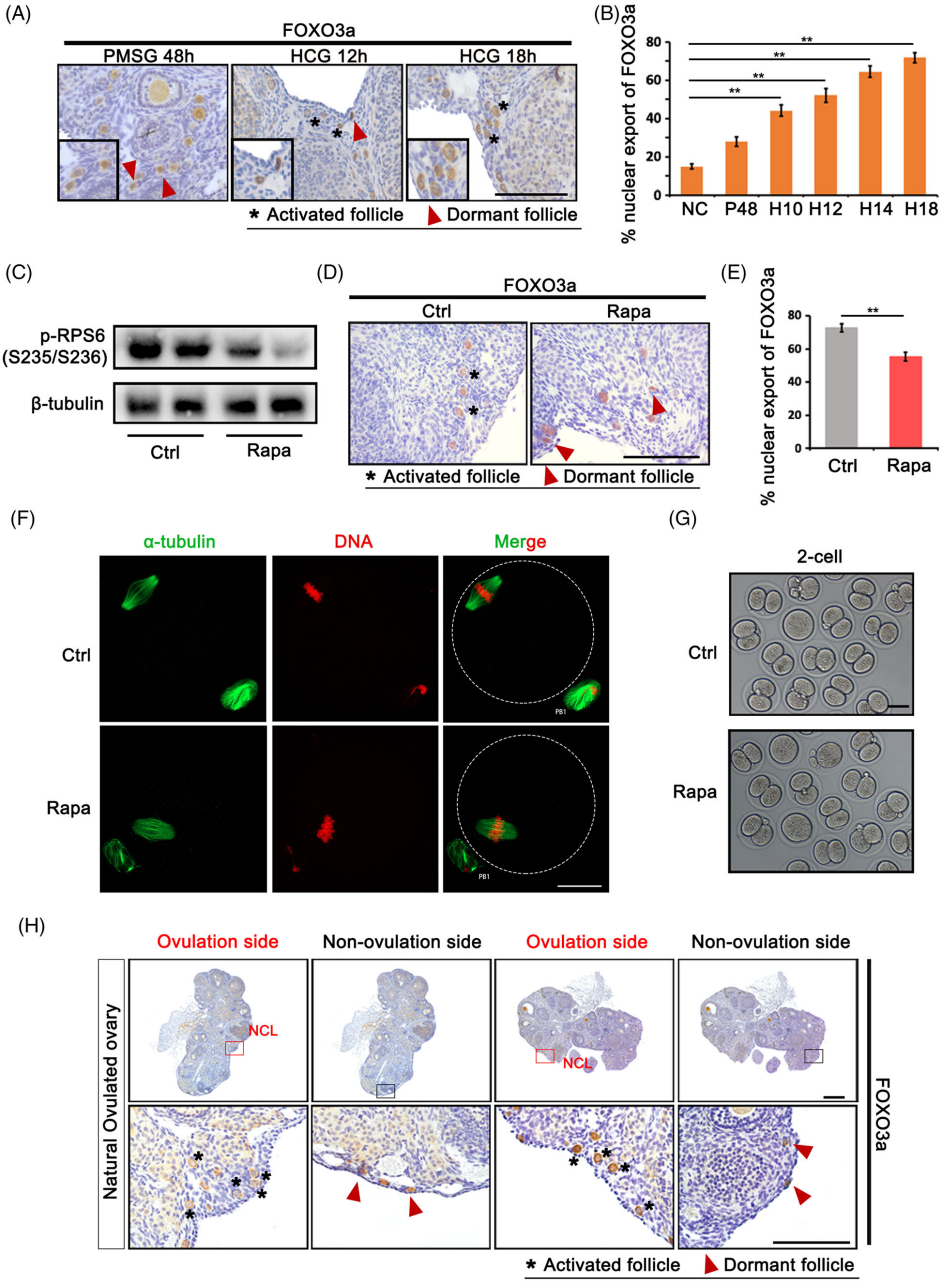
Ovulation stimulates the activation of primordial follicle through mTOR signalling pathway. Mice were superovulated by pregnant mare serum gonadotropin (PMSG) and human chorionic gonadotropin (HCG) treatment and ovaries were collected at indicated time points [negative control (NC), PMSG 48 h, HCG 10 h, HCG 12 h, HCG 14 h, HCG 18 h]. (A) Immunostaining of FOXO3a in primordial follicles at PMSG 48 h, HCG 12 h and HCG 18 h. FOXO3a shuttled from nuclei to cytoplasm with follicular activation. Black frame: representative magnified image with primordial follicles. (B) Percentage of activated primordial follicles at different time points in ovaries (NC, PMSG 48 h, HCG 10 h, HCG 12 h, HCG 14 h, HCG 18 h). (C) Blockage of p‐RPS6 (Ser235/236) by mTOR‐specific inhibitor rapamycin (Rapa) treatment. The expression of β‐tubulin was used as internal control. (D) Immunostaining of FOXO3a in primordial follicles in control and rapamycin‐treated ovaries. (E) Percentage of activated primordial follicles in control and rapamycin‐treated ovaries. (F) Immunofluorescence of α‐tubulin on spindle of MII oocytes in control and rapamycin group. Scale bars: 20 μm. (G) Representative two‐cell embryos after in vitro fertilisation (IVF). (H) Immunostaining of FOXO3a in primordial follicles under natural ovulation. Red square: ovulation site; black square: non‐ovulation site; NCL: new corpus luteum. Data represent the mean ± standard deviation (SD) of biological triplicate experiments. ^**^ *p* < .01, by one‐way analysis of variance (ANOVA) analysis. Scale bars: 50 μm

To evaluate the relationship between ovulation and follicular activation, ovaries were superovulated and collected at different time points [negative control (NC), pregnant mare serum gonadotropin (PMSG) 48 h, human chorionic gonadotropin (HCG) 10 h, HCG 12 h, HCG 14 h and HCG 18 h]. FOXO3a staining was used to label activated primordial follicles according to translocation from the oocyte nucleus to the cytoplasm after stimulation. As shown in Figure 1A,B, FOXO3a staining was limited in oocyte nucleus of most primordial follicles in both control non‐superovulated and PMSG‐treated ovaries; however, HCG treatment for 10 h initiated ovulation and corresponded with a significant increase in activated follicles (PMSG 48 h: 28%, HCG 10 h: 44%, HCG 14 h: 64%), and staining continued to increase until 18 h after HCG treatment (HCG 18 h: 72%). Our previous study has demonstrated HCG‐dependent activation of mTOR signalling pathway in PMSG‐primed mouse ovaries.^11^ To examine whether follicular activation caused by ovulation is related to this pathway, mice were injected with the mTOR inhibitor rapamycin (Rapa) 1 h before HCG treatment, and the results demonstrated the apparent inhibition of p‐RPS6 levels in the ovary after 18 h of HCG treatment (Figure 1C). The activated follicle counts according to FOXO3a staining further revealed the inhibitory effect of Rapa treatment on follicular activation (Figure 1D,E). Meanwhile, to exclude interference of Rapa on ovulation, we collected MII (metaphase II) oocytes for morphology and in vitro fertilisation (IVF) studies. The results showed comparable MII oocytes with normal meiotic spindles (α‐tubulin staining) and two‐cell embryonic development in Rapa‐treated mice (Figure 1F,G). Thus, Rapa treatment has no side effects on oocyte maturation and fertilisation, but inhibits primordial follicle activation induced by superovulation through the mTOR signalling pathway. Next, we collected naturally ovulated ovaries to check follicular activation by FOXO3a staining. Natural ovulation was guaranteed by monitoring the estrous cycle and observing ovulated oocytes in the ampullary segment of the oviduct. In the ovulated ovary, we could easily distinguish ovulated follicles according to the morphology of luteinising granulosa cells, and surrounding follicular activation was confirmed by the translocation of FOXO3a from the nucleus to the cytoplasm (Figure 1H, red square). However, primordial follicles distant from ovulated follicles remained dormant (Figure 1H, black square). Thus, limited follicular activation was induced by ovulation, which occurred in the local microenvironment.

### 2.2 Dynamic changes in polarised Mφs during ovulation

**FIGURE 2:**
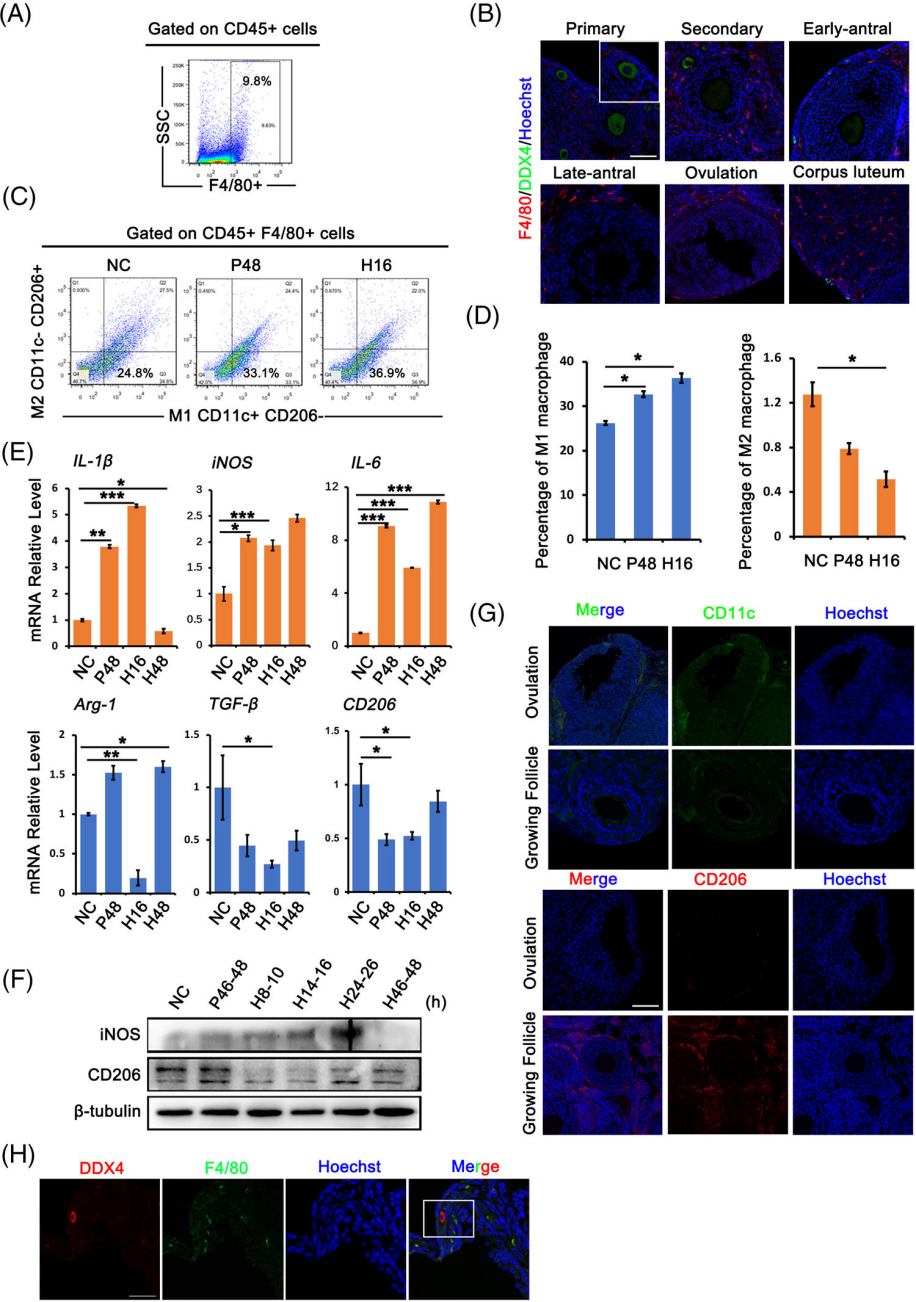
Dynamics of macrophages (Mφs) during ovulation. Mice were superovulated and ovaries were collected and divided into four phases [negative control (NC: diestrus, P48: follicle growing phase, H16: ovulation phase, H48: luteal phase]. (A) Fluorescence activated cell sorting analysis showed the content of Mφs in ovaries. Mφs were defined as CD45^+^ F4/80^+^ cells. (B) Immunofluorescence staining of F4/80 in follicles at different phases. Oocytes were labelled with DDX4 staining. Red: F4/80; green: DDX4; blue: Hoechst 33342. (C) Flow cytometry analysis of M1 and M2 Mφs at different follicle phases. M1 Mφs were defined as CD45^+^ F4/80^+^ CD11c^+^ CD206^−^ cells, M2 Mφs were defined as CD45^+^ F4/80^+^ CD11c^−^ CD206^+^ cells. (D) The proportion of M1 and M2 Mφs in ovaries at NC, pregnant mare serum gonadotropin (PMSG) 48 h and human chorionic gonadotropin (HCG) 16 h, respectively. (E) Relative expression of M1 Mφs markers (*IL‐1β*, *iNOS*, *IL‐6*) and M2 Mφs markers (*Arg‐1*, *TGF‐β*, *CD206*) in ovaries at each follicle phase. (F) Western blot analysis of iNOS and CD206 expression in ovaries at different follicle phases. The expression of β‐tubulin was used as internal control. (G) Immunofluorescence labelling of CD11c and CD206 in ovulated follicles and growing follicles in ovaries collected at 16 h after HCG treatment. Green: CD11c; red: CD206; blue: Hoechst. (H) Aggregates of Mφs (F4/80) around primordial follicles near ovulated follicles. Ovaries were collected at 16 h after HCG treatment. Oocytes were labelled with DDX4. Red: DDX4; green: F4/80; blue: Hoechst 33342. Data represent the mean ± standard deviation (SD) of biological triplicate experiments. ^*^ *p* < .05, ^**^ *p* < .01 and ^***^ *p* < .001, by one‐way analysis of variance (ANOVA) analysis. Scale bars: 50 μm

The important roles of Mφs in follicular development and ovulation have been widely reported; however, because different polarised Mφs function differently in inflammation, it remains unclear how Mφ state is regulated during these processes. We first collected adult (8 weeks) mouse ovaries for quantification by flow cytometry, and the results revealed that Mφs (CD45^+^, F4/80^+^) accounted for approximately 10% of all cells in the ovary (Figure 2A). Immunofluorescence staining with F4/80 revealed that Mφs were mainly localised in the ovarian interstitium, corpus luteum and theca, and almost all stages of follicles, including primary, secondary, antral and ovulated follicles, were surrounded by scattered Mφs (Figure 2B). We then quantified M1 and M2 Mφs in superovulated ovaries collected at different time points (NC, PMSG 48 h, HCG 16 h) by flow cytometry and the result demonstrated that M1 Mφs are the primary subtype in the ovary (Figure 2C). In control non‐treated ovaries (NC group), M1 Mφs accounted for 26% of total Mφs, whereas only 1.2% were M2 Mφs. After superovulation with PMSG and HCG treatment, Mφs counts changed dynamically, with significant increase of M1 Mφs (36%) and dramatic decrease of M2 Mφs (0.5%) in HCG‐treated ovaries (Figure 2D). Consistently, reverse transcription‐polymerase chain reaction (RT‐PCR) data revealed the increased expression of the pro‐inflammatory genes *IL‐1β*, *iNOS* (inductible Nitrix Oxide Synthase) and *IL‐6* and the markedly decreased expression of the M2 marker genes *Arg‐1*, *TGF‐β* and *CD206* in ovaries after HCG treatment (Figure 2E). Western blot with iNOS and CD206 also showed a gradual increase in iNOS expression after HCG treatment, whereas the expression of CD206 decreased significantly during ovulation. However, the expression of both iNOS and CD206 was restored to non‐treated status (NC) after 48 h of HCG treatment when the corpus luteum formed (Figure 2F). Immunostaining for CD11c and CD206 in the ovulated ovary (14 h after HCG treatment) showed the differential distribution of M1 and M2 Mφs, with M1 Mφs mainly localised around ovulated follicles and M2 Mφs around growing follicles (Figure 2G). Especially, we noticed the aggregation of M1 Mφs around primordial follicles near ovulated follicles (Figure 2H). Thus, dynamic changes in local Mφs have the potential to regulate follicular activation.

### 2.3 Differential regulation of follicular activation by Mφ subtypes

**FIGURE 3:**
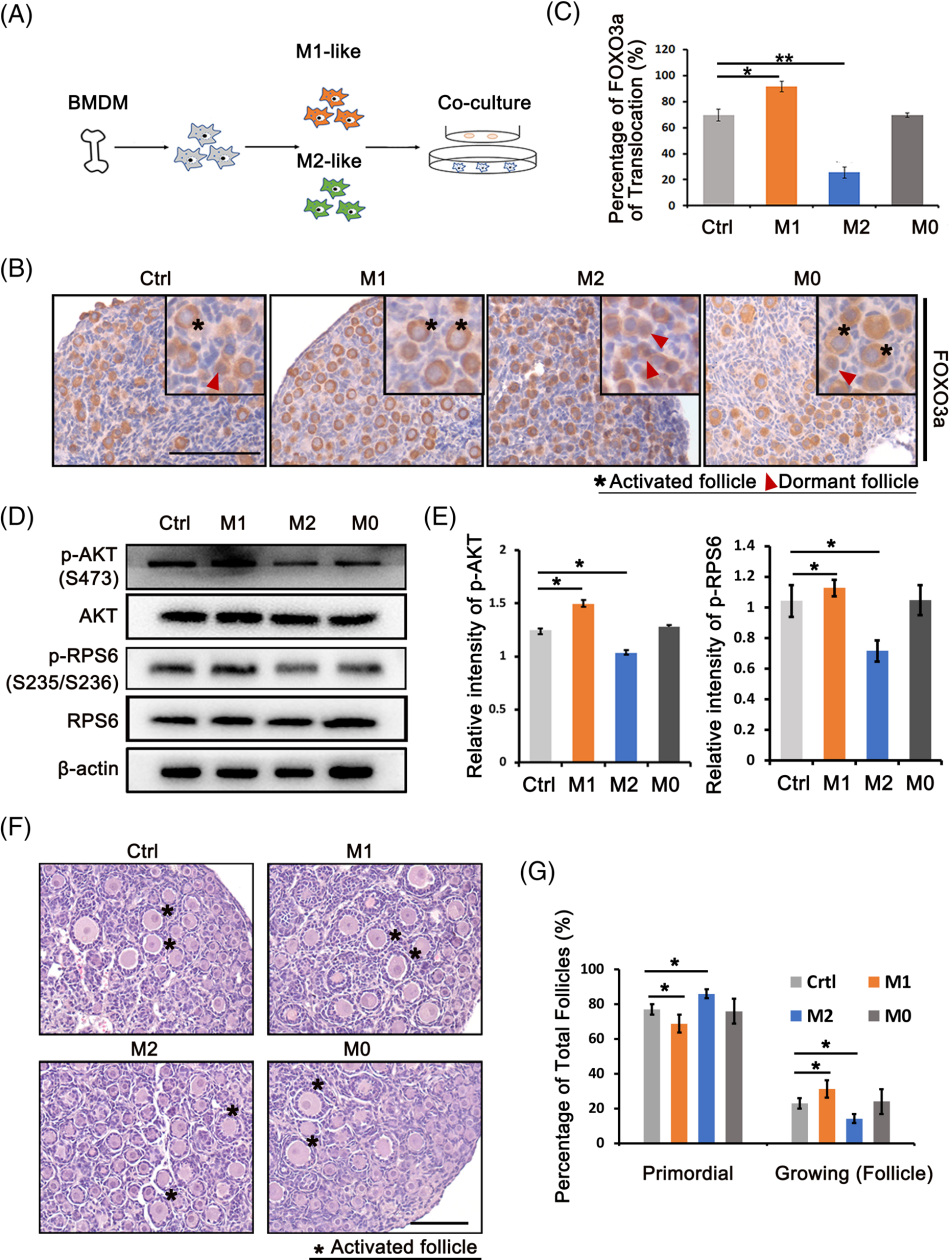
The differential regulation of M1 and M2 macrophages (Mφs) on follicular activation. Bone marrow‐derived macrophages (BMDMs) were isolated from mouse femur and tibia bones and polarised into M1 and M2 Mφs by LPS and interleukin‐4 (IL‐4), respectively. P2.5 ovaries were cocultured with M0, M1 and M2 Mφs for 24 h, respectively. (A) Schema for BMDM and ovary coculture system. (B) Immunostaining of FOXO3a in ovaries collected from control, M1, M2 and M0 groups. (C) Percentage of activated primordial follicles in control, M0, M1 and M2 groups, respectively. (D) Western blot analysis of PI3K/mTOR signal‐related proteins, p‐AKT (Ser473) ad p‐RPS6 (Ser235/236) in ovaries of different groups. The expression of AKT, RPS6 and β‐actin were used as internal controls. (E) Expression intensity of p‐AKT and p‐RPS6. (F) Histology of ovaries collected at 96 h in each group. After 24 h of treatment, ovaries in each group were further cultured in control media for 72 h. Asterisk indicates growing follicle. (G) Distributions of primordial and growing follicles in ovaries collected after 96 h of culture. Data represent the mean ± standard deviation (SD). of biological triplicate experiments. ^*^ *p* < .05, ^**^ *p* < .01 as compared with controls, by one‐way analysis of variance (ANOVA) analysis. Scale bars: 50 μm

To determine the effects of Mφs on primordial follicles, we established an in vitro coculture model in which newborn ovaries (P2.5) were incubated with Mφs in different polarised states (Figure 3A). Primary bone marrow‐derived Mφs (BMDM) with high purity were polarised into M1 and M2 Mφs by LPS and interleukin‐4 (IL‐4) treatment, respectively (Figure S1A,B). The properties of M1 and M2 Mφs were confirmed by immunofluorescence and RT‐PCR (Figure S1C,D). After 24 h of coculture, ovaries in each group were collected for FOXO3a staining. There was no difference in activated follicle count between the M0 group and the control group. However, an increased number of activated primordial follicles was observed in the M1 group compared with the control group, whereas the M2 group showed the inhibition of follicular activation (Figure 3B,C). Western blotting was then performed to investigate the effects on the PI3K/mTOR pathway, and the results showed increased levels of p‐AKT (phosphorylated AKT serine/thereonine kinase), the downstream effector of the PI3K signalling pathway, and p‐RPS6, the downstream effector of the mTOR signalling pathway, in M1‐treated ovaries, whereas the levels of both phosphorylated proteins were markedly decreased in the M2 group (Figure 3D,E). We also examined the effects of different Mφs on cellular apoptosis, and no obvious difference was observed after short‐term coculture (Figure S1E). Next, after coculture with Mφs, the ovaries in each group were further cultured in control medium for 72 h to evaluate follicular growth and development. Histology revealed accelerated follicular development in M1‐treated ovaries, with 31% growing follicles compared with 23% and 24% in control and M0‐treated ovaries, respectively (Figure 3F,G). However, only 14% of follicles in ovaries treated with M2 Mφs were detected in the growth state. The results indicated that Mφs are related to primordial follicle activation, which is promoted by M1 Mφs and reduced by M2 Mφs.

### 2.4 Mφ‐derived EVs participate in the differential regulation of primordial follicle activation

**FIGURE 4:**
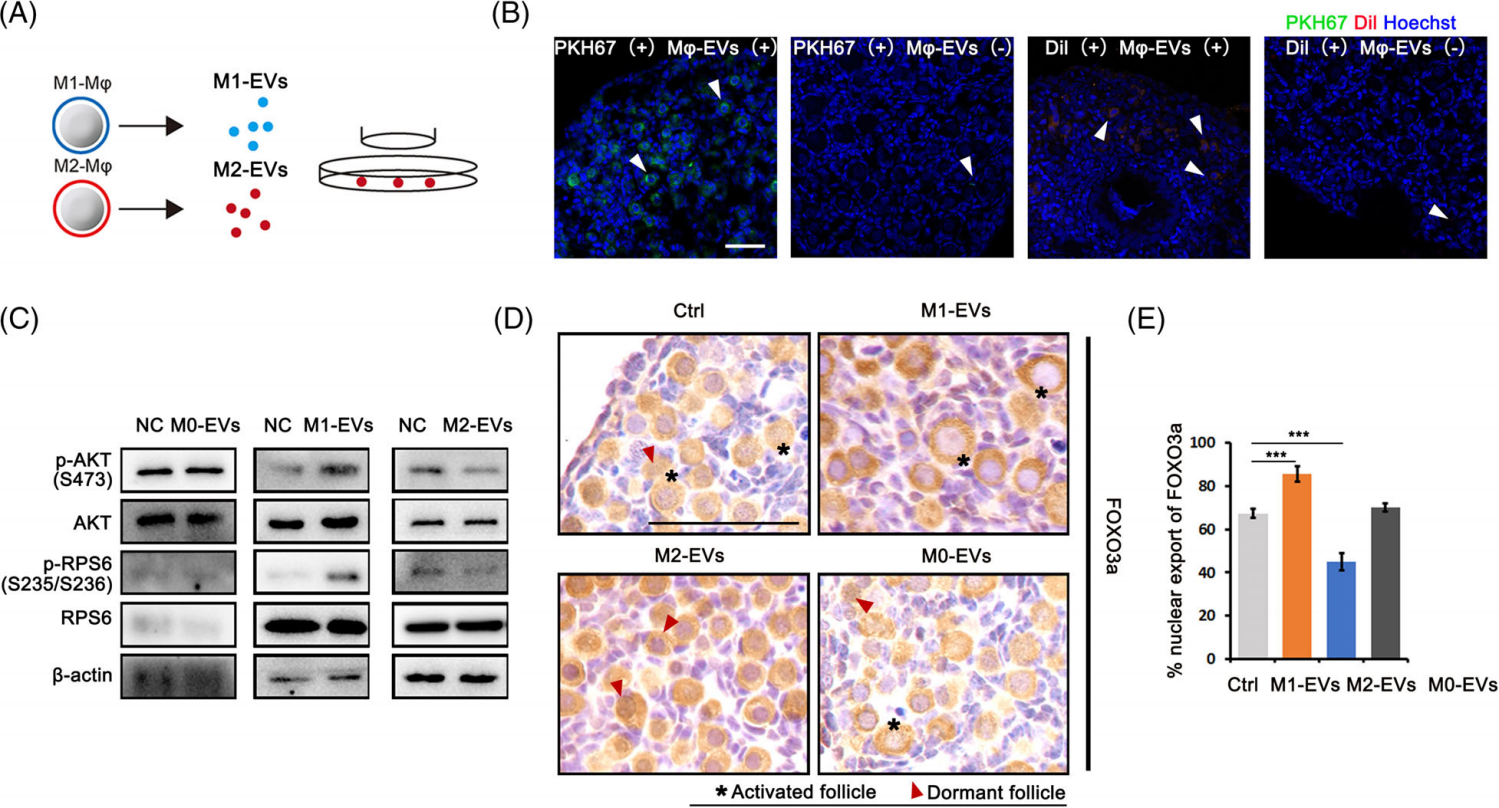
Macrophage‐derived extracellular vesicles (EVs) affect activation of primordial follicles. EVs were isolated from M0, M1 and M2 macrophages (Mφs), respectively. P2.5 ovaries were cocultured with M0‐, M1‐ and M2‐EVs at 1.9 × 10^11^ particles/ml for 24 h. (A) Schema for Mφ‐derived EVs and ovary coculture system. (B) Accumulation of PKH67‐labelled EVs in primordial follicles. Ovaries were collected for frozen sections and nuclei were stained with Hoechst 33342 (green: EVs; blue: Hoechst 33342). (C) Ovarian expression of PI3K/mTOR signal‐related proteins p‐AKT (Ser473) and p‐RPS6 (Ser235/236) in ovaries cocultured with Mφ‐derived EVs. The expression of AKT, RPS6 and β‐actin were used as internal control. (D) Immunostaining of FOXO3a in primordial follicles in each group after 24 h of culture. (E) Percentage of activated primordial follicles in ovaries of each group. Data represent the mean ± standard deviation (SD) of biological triplicate experiments. ^***^ *p* < .001, by one‐way analysis of variance (ANOVA) analysis. Scale bars: 50 μm

Recently, an increasing number of studies have reported the regulatory role of Mφ‐derived EVs on target cellular functions. According to the phenotypes of Mφs, there are three main types of Mφ‐derived EVs, unpolarised M0 Mφ‐derived EVs (M0‐EVs) and polarised M1 and M2 Mφ‐derived EVs (M1‐EVs and M2‐EVs), that have been reported to reflect their parental cell functions. Since no direct contacts were found between Mφs and primordial follicles (Figure 2H), we investigated whether Mφ‐derived EVs participate in follicular activation (Figure 4A). M0‐, M1‐ and M2‐EVs were isolated from their parent cells, and the identity of representative M0‐EVs was shown by particle size, transmission electron microscopy (TEM) and surface marker expression (Figure S1F–H). The ability of ovaries to take up EVs was then evaluated by incubating PKH67‐ or Dil‐labelled M0‐EVs with newborn ovaries for 24 h, and the results showed the specific enrichment of EVs in primordial oocytes (Figure 4B). Next, newborn ovaries were incubated with M0‐, M1‐ and M2‐EVs for 24 h, and ovaries were collected for western blotting and immunostaining. Figure 4C showed that M0‐EVs had no effect on activation of the PI3K/mTOR signalling pathway. The levels of phosphorylated AKT and phosphorylated RPS6 in the ovary were increased by M1‐EVs but significantly reduced by M2‐EVs. FOXO3a staining and the activated follicle count revealed an increased number of activated primordial follicles in the M1‐EVs group, whereas less activation was observed in M2‐exo‐treated ovaries (Figure 4D,E). These results indicated that like their parent cells, polarised Mφ‐derived EVs affected primordial follicle activation.

### 2.5 Mφ‐EVs contained miRNAs that differentially regulate Mφ subtypes during primordial follicle activation

**FIGURE 5:**
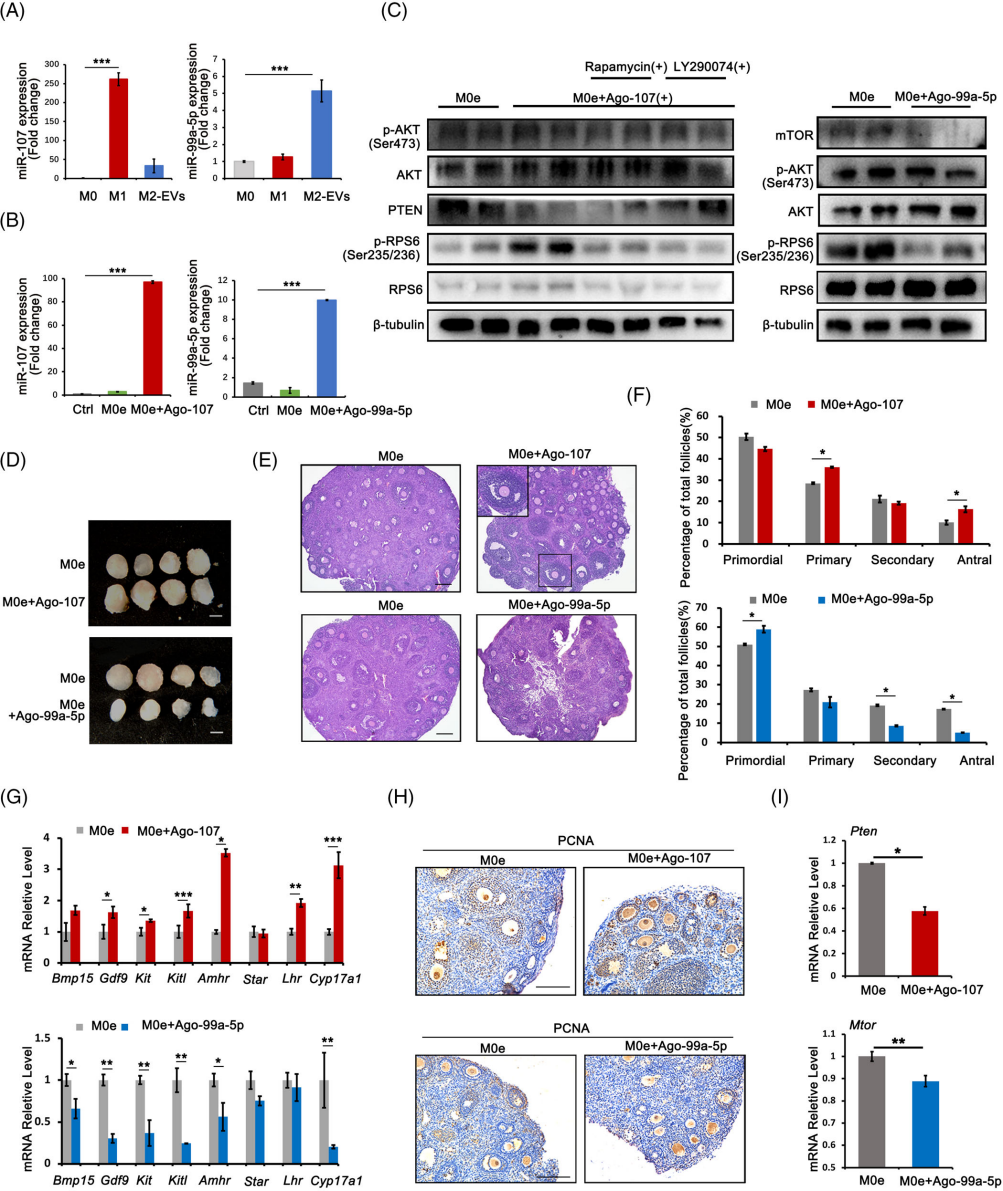
Extracellular vesicles (EVs)‐carried microRNAs participated in the regulation of macrophages (Mφs) on follicular activation and development. (A) Expression of miR‐107 and miR‐99a‐5p in M0, M1 and M2 Mφs‐derived EVs. (B) Expression of miR‐107 and miR‐99a‐5p in ovaries after different treatments. Ctrl: phosphate‐buffered saline (PBS); M0e: M0‐EVs; M0e + Ago‐107: M0‐EVs with agomir‐107; M0e + Ago‐99a‐5p: M0‐EVs with agomir‐99a‐5p. (C) Ovarian expression of p‐AKT (Ser473) and p‐RPS6 (Ser235/236) after 24 h of treatment. In M0e + Ago‐107 group, mTOR inhibitor rapamycin (Rapa) and PI3K inhibitor LY290074 was pretreated for 1 h and the protein levels of PTEN, the miR‐107 target was checked. In M0e + Ago‐99a‐5p group, the protein levels of mTOR, the target of miR‐99a‐5p was examined. The expression of AKT, RPS6 and β‐tubulin were used as internal control. (D) Comparisons of ovarian development after various treatment combinations. Paired ovaries were separated and treated with M0e or M0e + Ago‐107 and M0e or M0e + Ago‐99a‐5p, respectively, for 24 h. Paired ovaries with different treatments were then transplanted under kidney capsules of recipient mice for 14 days. Scale bar: 1 mm. (E) Ovarian histology between paired ovaries and distribution of different stages of follicles in paired ovaries treated with M0e and M0e + Ago‐107 and with M0e and M0e + Ago‐99a‐5p. Black frame: representative image of antral follicle. (F) Follicles counts revealed the percentage of follicles at different developmental stages in each treatment group. (G) Relative expression of follicular growth and development‐related genes in ovaries of each group. The levels of all tested mRNAs in M0e group were set to 1. (H) Ovarian proliferating cell nuclear antigen (PCNA) staining in nuclei of oocytes and granulosa cells in various groups. (I) Relative expression of in M0e + Ago‐107 and M0e + Ago‐99a‐5p‐treated ovaries, respectively. The expression of *PTEN* and *mTOR* mRNAs in M0e group were set to 1. Data represent the mean ± standard deviation (SD) of biological triplicate experiments. ^*^ *p* < .05, ^**^ *p* < .01, ^***^ *p* < .001, by one‐way analysis of variance (ANOVA) analysis. Scale bars: 50 μm

EVs have been reported to function through their cell‐specific proteins, lipids and RNAs, including mRNAs, miRNAs and other noncoding RNAs. Previous studies identified the differential expression of EVs miRNAs in Mφ subtypes, and these specific EVs miRNAs functioned as expected based on their parent cells in multiple developmental processes and disease models.^24^ To elucidate the underlying mechanism of the two types of Mφ‐derived EVs on primordial follicles, we considered the published miRNA GEO database to identify candidates.^25^, ^26^ Considering the differential regulation of primordial follicle activation by M1 and M2 Mφs and their effects on the PI3K/mTOR signalling pathway, we selected two EVs‐specific miRNAs, the M1‐EVs‐specific miR‐107 and the M2‐EVs‐specific miR‐99a‐5p, for further analysis. Both miRNAs have been previously reported to participate in regulating the PI3K/mTOR signalling pathway.^27^, ^28^, ^29^, ^30^ We first validated the specific expression of the two miRNAs in M1‐ and M2‐EVs by RT‐PCR (Figure 5A). Since M0‐EVs had no effect on primordial follicles, we transferred miRNA mimics agomir‐107 and agomir‐99a‐5p into M0‐EVs to demonstrate their functions in follicular activation. After 24 h of incubation, RT‐PCR showed significant increases in miR‐107 and miR‐99a‐5p‐treated ovaries (Figure 5B). Together, we also detected the significant decreased expression of miR‐107 target PTEN and miR‐99a‐5p target mTOR in treated ovaries, respectively (Figure 5C,I). Western blot then revealed the stimulatory effects of agomir‐107 on the levels of p‐AKT and p‐RPS6, which could be effectively blocked by the pretreatment with PI3K inhibitor LY290074 and mTOR inhibitor Rapa, whereas agomir‐99a‐5p inhibited the phosphorylation of these two PI3K/mTOR downstream effectors (Figure 5C). Paired ovaries treated with different agomirs were then used for kidney capsule transplantation to evaluate the effects of the two agomirs on primordial follicle activation and follicular development. Compared with control ovaries, those treated with agomir‐107 increased in size, while those treated with agomir‐99a‐5p got smaller (Figure 5D). Paired ovaries were then collected for morphological analysis and follicle counts. As shown in Figure 5E, agomir‐107‐treated ovaries had a higher percentage of growing follicles, whereas follicular development was inhibited in agomir‐99‐5p‐treated ovaries, with more primordial follicles and fewer growing follicles (Figure 5F). Moreover, the RT‐PCR results indicated that the expression of the oocyte development‐related genes *Bmp15*, *Gdf9* and *Kit*; the granulosa cell‐associated genes *Amhr* and *Kitl*; and the gonad steroid synthesis‐related genes *Star*, *Lhr* and *Cyp17a1* was higher in agomir‐107‐treated ovaries but significantly lower in agomir‐99a‐5p‐treated ovaries (Figure 5G). Additionally, proliferating cell nuclear antigen (PCNA) staining showed increased positive signals in the nuclei of oocytes and granulosa cells in the agomir‐107 group and decreased positive signals in the agomir‐99a‐5p group (Figure 5H). Taken together, these results suggested the specific effects of M1‐ and M2‐EVs‐derived miRNAs on follicular activation and development.

### 2.6 In vivo treatment with M2‐EVs improved ovarian function in old mice

Chronic, sterile and low‐grade inflammation has been observed in aged ovaries.^31^ Previous studies have revealed the link between Mφs, especially M1 Mφs, and ovarian ageing; however, since relatively fewer M2 Mφs are detected in ovaries, the role of these cells in the ageing process is intriguing. Based on our findings regarding the differential regulation of primordial follicles by M1 and M2 Mφs and their derived EVs, we next explored their effects on ovarian ageing. The increased inflammation in aged ovaries was first manifested by elevated expression of the pro‐inflammatory genes *TNF‐α*, *IL‐6*, *iNOS* and *IL‐17* and the inflammasome‐related genes *Asc* and *Nlrp3*. Meanwhile, compared with control young ovaries, the anti‐inflammatory genes *IL‐10* and *Arg‐1* but not *CD206* were significantly downregulated in aged ovaries (Figure S2A). We then compared the proportion phenotype of Mφs in young and aged ovaries by flow cytometry. The result demonstrated significantly increased M1 in aged ovaries, however, no statistical difference was found in M2 Mφs between control and aged ovaries (Figures S2B and 2C). Western blot and immunostaining further revealed increased expressions of M1 Mφs markers iNOS and CD11c in aged ovaries (Figure S2D–F). The above results suggested a pro‐inflammatory environment during ovarian ageing.

**FIGURE 6:**
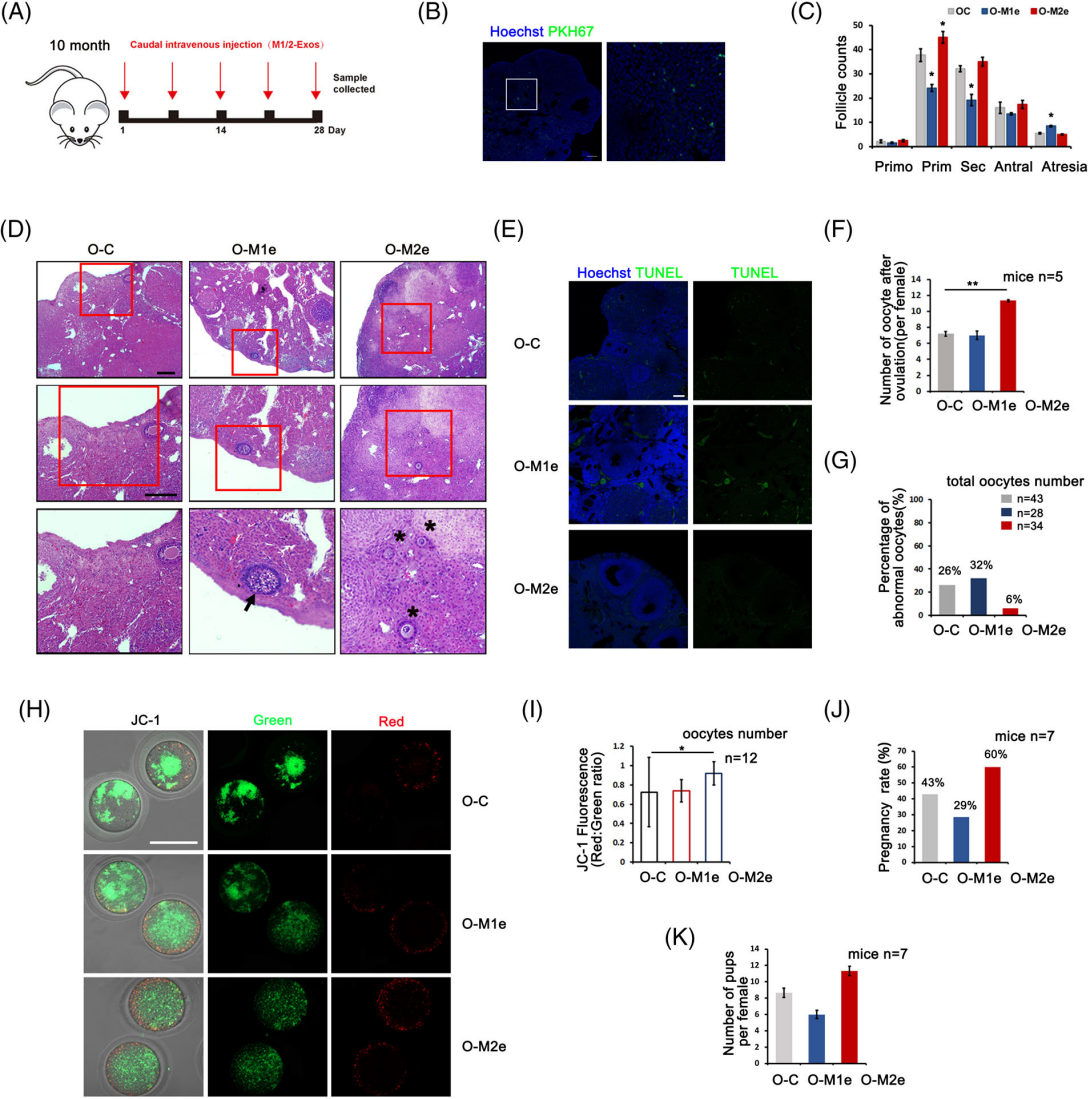
Improvement of ovarian function after M2‐extracellular vesicles (EVs) treatment in aged mice. Mice at 10‐month of age were tail intravenous injected with 100 μl phosphate‐buffered saline (PBS) containing 15 μg M1‐, M2‐EVs (O‐M1e and O‐M2e) and same volume of PBS (O‐C) five times for 28 days, respectively. (A) Schema for in vivo macrophage (Mφ)‐EVs treatment. (B) Fluorescence localisation of PKH67‐labelled Mφ‐EVs in ovary. Green: EVs; blue: Hoechst 33342. (C) Distribution of different stages of follicles in each group. Primo: primordial follicle; Prim: primary follicle; Sec: secondary follicle; Antral: antral follicle; Atresia: atretic follicle. (D) Representative follicles in O‐C, O‐M1e and O‐M2e ovaries. Middle and down panels are higher magnifications of up panels. Arrow: atretic follicle; asterisk: primary follicle. (E) TUNEL staining to detect apoptosis in ovaries of each group. Green: TUNEL signal; blue: Hoechst 33342. (F) Numbers of ovulated oocytes in each group. *n* = 5 mice in each group. (G) Percentage of oocyte abnormality in each group. Oocyte number in each group was labelled. (H) Mitochondrial membrane potential shown by JC‐1 staining in oocytes of three groups. *n* = 10 oocytes in each group. (I) The ratio of red/green fluorescence intensity reflects the oocyte mitochondrial ∆Ψm in each group. Oocytes *n* = 12. (J) Pregnancy rate of O‐C, O‐M1e and O‐M2e‐treated mice after mating with male mice. *n* = 7 mice in each group. (K) Average number of pups in the first litter after mating. *n* = 7 mice in each group. Data represent the mean ± standard deviation (SD) of biological triplicate experiments. ^*^ *p* < .05, ^**^ *p* < .01, by one‐way analysis of variance (ANOVA) analysis. Scale bars: 50 μm

In the following experiments, we intravenously injected phosphate‐buffered saline (PBS), M1‐ or M2‐EVs into aged mice (10 months old) and observed the effects on ovarian function. The procedure is shown in Figure 6A, and mice received a total of five injections of EVs over 28 days. PKH67‐labelled Mφ‐EVs were first used for EVs‐tracing, and localisation in the ovary was observed at the end of the procedure (Figure 6B). Ovaries in each group were then collected for morphology and follicle counting. Compared with PBS‐treated ovaries (O‐C), the M1‐EVs‐treated ovaries (O‐M1e) showed extreme damage in terms of follicular development, including a decreased follicle count at all developmental stages and an increase in atretic follicles. In contrast, M2‐EVs‐treated ovaries (O‐M2e) showed an obvious increase in the number of growing follicles and a reduction in follicular atresia (Figures S3A and 6C,D). TUNEL (TdT‐mediated dUTP Nick‐End Labeling) assay further demonstrated increased apoptosis in M1‐EVs‐treated ovaries (Figure 6E) while PCNA staining showed increased cell proliferation in M2‐EVs‐treated ovaries (Figure S3B). The recovery of ovarian function was also manifested by the raised gene expressions related with follicular development and the elevated serum AMH (Anti‐Mullerian Hormone) and E2 levels in M2‐EVs‐treated mice (Figure S3C,D). We next evaluated oocyte quality after superovulation, and the results showed that M2‐EVs‐treated mice produced more oocytes than those in the other two groups (11.3 oocytes/mouse in the O‐M2e group vs. 7.2 and 7.0 oocytes/mouse in the O‐C and O‐M1e groups) (Figure 6F). The analysis of oocyte morphology showed a high proportion of abnormalities among PBS‐ and M1‐EVs‐treated oocytes, especially in M1‐EVs‐treated oocytes, with an even higher proportion of fragmented oocytes (32% in the O‐M1e group vs. 26% in the O‐C group) (Figure 6G). However, we only detected 6% fragmented oocytes in the O‐M2e group. Oocyte mitochondrial transmembrane potential (∆Ψm) was then measured through a ratiometric analysis of JC‐1 staining, and the increased red/green fluorescence ratio indicated an increase in oocyte mitochondrial activity after M2‐EVs treatment (Figure 6H,I). The results suggested that M2‐EVs treatment not only increased oocyte production, but also improved oocyte quality. We also evaluated the fertility of treated mice in each group by a natural mating experiment. The first delivery was estimated and compared with PBS‐treated mice (43% pregnancy rate), M2‐EVs‐treated mice showed a 60% pregnancy rate, with an average delivery of 11 pups/mouse; however, M1‐EVs‐treated mice had a worse pregnancy rate (29%), and delivered only 5 pups/mouse (Figure 6J,K). Taken together, these results demonstrated the ability of M2‐EVs to improve ovarian function in aged mice.

### 2.7 M2‐EVs function by regulating the immune microenvironment in ageing ovaries

**FIGURE 7:**
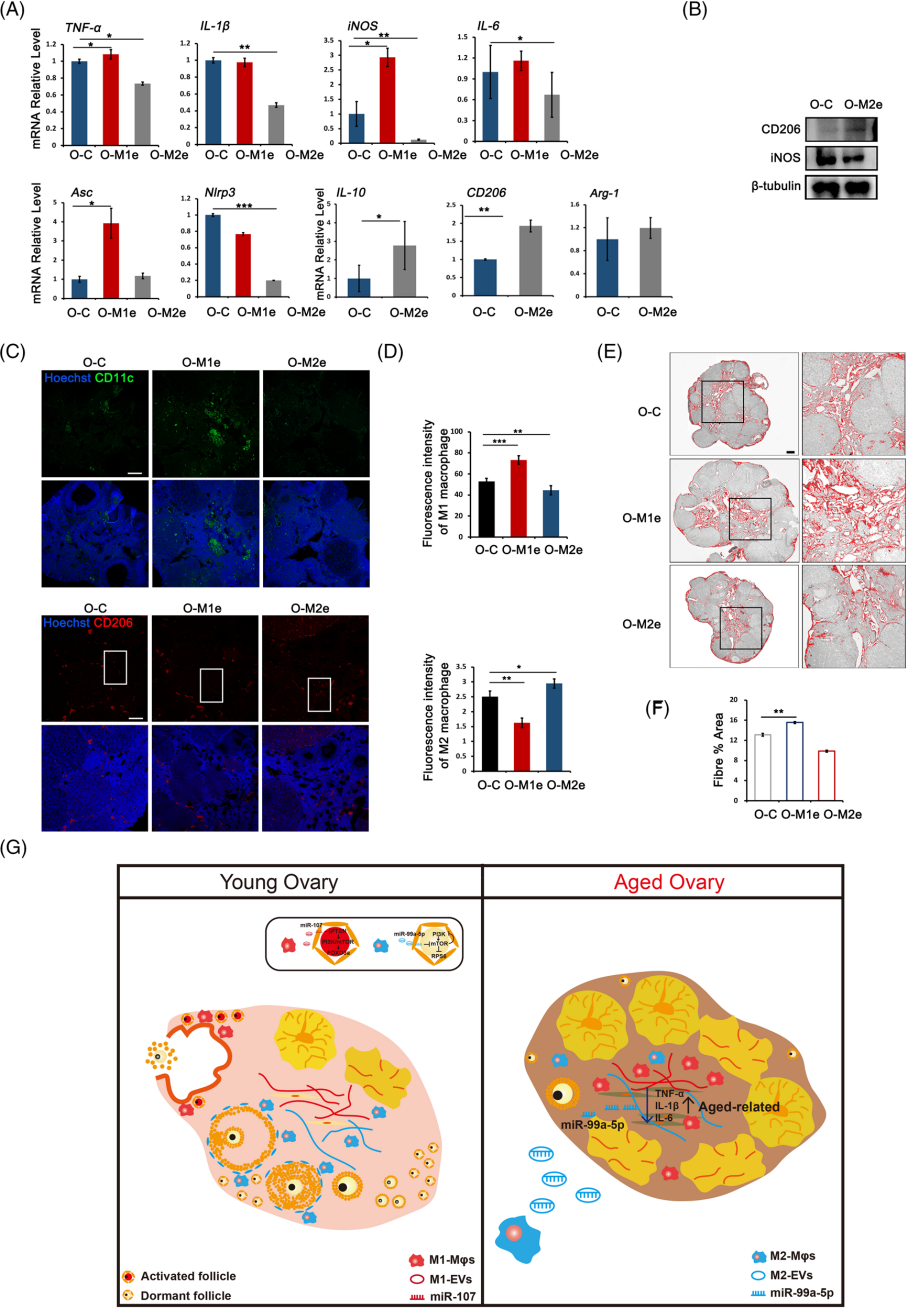
M2‐extracellular vesicles (EVs) changed inflammatory microenvironment in aged ovaries. Mice at 10‐month of age were tail intravenous injected with 100 μl phosphate‐buffered saline (PBS) containing 15 μg M1‐, M2‐EVs (O‐M1e and O‐M2e) and same volume of PBS (O‐C) five times for 28 days, respectively. (A) The expression of pro‐inflammatory genes and anti‐inflammatory genes in aged ovaries of three groups. (B) Western blot of iNOS and CD206 expression in O‐C and O‐M2e ovaries. The expression of β‐tubulin was used as internal control. (C) Immunofluorescence labelling of CD11c and CD206 in aged ovaries treated with PBS, M1‐EVs and M2‐EVs. (D) Fluorescent intensity of CD11c and CD206 in ovaries of each group. (E) Representative processed colour threshold images of Masson‐stained ovarian tissue sections in ovaries of different groups. Right panels represent the magnified image of black frames. (F) Quantification of ovarian fibrosis by Masson‐stain after mice were treated with PBS, M1‐ or M2‐EVs. (G) A schematic diagram depicts the role of macrophages (Mφs) in regulating primordial follicle activation after physiological ovulation and ovarian inflammatory environment during ovarian ageing. Left panel: an inflammatory environment consisting of M1 Mφs that gather around the ovulation site stimulates primordial follicles activation in each menstrual cycle. Right panel: the accumulation of M1 Mφs in aged ovaries induces a pro‐inflammatory environment and reduced ovarian function. M2‐EVs treatment alters the inflammatory microenvironment and improves ovarian function in aged ovaries at least partly through M2‐EVs contained miRNA, miR‐99a‐5p. Data represent the mean ± standard deviation (SD) of biological triplicate experiments. ^*^ *p* < .05, ^**^ *p* < .01 and ^***^ *p* < .001, by one‐way analysis of variance (ANOVA) analysis. Scale bars: 50 μm

We next evaluated whether the beneficial effect of M2‐EVs on the aged ovary was related to their anti‐inflammatory functions. Real‐time PCR results showed that M2‐EVs treatment decreased the expression of pro‐inflammatory genes, including *TNF‐α, IL‐6*, *iNOS* and *IL‐1β*, but significantly increased the expression of the anti‐inflammatory genes *IL‐10*, *Arg‐1* and *CD206* (Figure 7A). Western blot analysis showed that M2‐EVs downregulated the expression of the M1 Mφ‐related protein iNOS but upregulated the M2 Mφ marker CD206 (Figure 7B). The distribution of Mφ subtypes was then detected by CD11c and CD206 immunostaining, and the results showed decreased positivity for M1 Mφs but increased positivity for M2 Mφs in M2‐EVs‐treated ovaries (Figure 7C,D). Increased collagen deposition and fibrosis can be the result from chronic inflammation; therefore, we assessed ovarian fibrosis by Masson staining after treatment with M1‐ or M2‐EVs. As shown in Figure 7E,F, fibrosis mainly occurred in the ovarian interstitial area. Compared with the ovaries of PBS‐injected control mice, those of M1‐EVs‐treated mice showed a significant increase in the positive area of fibrosis. However, fibrosis was not greatly affected in M2‐EVs‐treated ovaries. RT‐PCR on ovarian stromal cells collected from three groups of mice further demonstrated the significantly increased expression of fibrosis‐related genes *Col1a1*, *Col1a2* and *α‐SMA* in M1‐EVs group but not in M2‐EVs group (Figure S4A). These results suggest that M2‐EVs regulate the ovarian inflammatory environment without affecting ovarian fibrosis. Finally, we isolated primary stroma cells from aged mice and treated with M0‐EVs and M0‐EVs transfected with agomir‐99a‐5p for 24 h to explain how M2‐EVs reduced inflammation in aged ovaries. The property of stroma cells was first verified by the lower expression of granulosa marker *Fshr* and oocyte marker *Bmp15*. Western blot then demonstrated the inhibition of PI3K/mTOR signalling pathway and the reduction of α‐SMA proteins in stroma cells treated with M0‐EVs contained agomir‐99a‐5p. RT‐PCR also showed the decreased expression of *IL‐6* and *IL‐1β* in the agomir‐treated group (Figure S4B–D). Taken together, the results suggest that M2‐EVs treatment may alter the inflammatory microenvironment in the aged ovary through its downregulation on the mTOR signalling pathway by contained specific miRNAs.

## 3 DISCUSSION

It has long been accepted that the activation of primordial follicles is gonadotropin‐independent and that the primordial‐to‐primary transition is chiefly regulated by regulatory factors in the surrounding environment.^32^ Until now, it was unclear why only a small number of primordial follicles are recruited into the growing follicle pool, while most follicles remain dormant for decades. In the current study, mice treated with PMSG or HCG showed a gradual increase in primordial follicle activation in response to ovulation. Additionally, in naturally ovulated ovaries, we found selective follicular activation near the ovulated follicles. This is consistent with what we reported previously about the differential activation of primordial follicles near or distant from the incision site after ovarian partial resection.^11^ We thus propose that ovulation is the motivation for selective follicle recruitment in each menstrual cycle. The ovulatory process, akin to a wound, ending with rupture of the follicular wall and expulsion of the oocyte, is preceded by the inflammatory reactions that occur in mature follicles.^33^ The injury‐induced primordial follicle activation can be explained by accelerated follicle recruitment after ovarian intervention.^34^ For example, in PCOS‐induced anovulation, wedge resection or ovarian ‘drilling’ is a valid procedure to induce follicular development^35^; in women with endometriomas, cystectomy induces an immediate decline in antral follicle count, but partial restoration is normally observed several months later.^36^ One recent study reported that mice exposed to repeated superovulation showed an obvious decrease in the primordial follicle pool and poor oocyte quality.^37^ Another study reported ovulation suppression protected against egg aneuploidy in aged female mice.^38^ Although the effects of repeated assisted reproduction techniques on ovarian response in humans remain controversial,^39^ our findings suggest that the increased risk of repetitive ovarian stimulation on ovarian reserve should be considered in the clinic. In this meaning, ovulation suppression could also be a good way to protect primordial follicle reserve and delay ovarian ageing. Since a single injection of the mTOR inhibitor rapamycin before HCG treatment showed the inhibition of follicular activation but without any side effects on ovulation or fertilisation, rapamycin may be another candidate to preserve primordial follicles from overstimulation induced by internal or external inflammatory factors.

As the most abundant immune cells in the ovary, Mφ has been reported to play multiple roles in follicle growth, atresia, ovulation, and corpus luteum formation and regression.^40^, ^41^ Studies using CD11b, CD11c or CD206 diphtheria toxin receptor transgenic mice revealed the important role of CD11c^+^ M1 Mφs in regulating folliculogenesis. Like the phenotypes revealed by CD11b^+^ pan‐Mφ ablation, CD11c^+^ M1 Mφ deletion in the ovary resulted in follicle atresia and bleeding, whereas CD206^+^ M2 Mφ‐depleted mice exhibited normal follicular development and ovulation.^20^ Just prior to ovulation, an increased number of Mφs were found in the interstitial‐theca layers of preovulatory follicles, and rapid and extensive infiltration of Mφs occurs upon induction of the LH surge.^42^ In this study, the significant increase in M1 Mφs during ovulation was verified by RT‐PCR, western blotting and flow cytometry, and these findings were consistent with the increased follicular activation in HCG‐primed ovaries. Compared with the M1 Mφ count, the M2 Mφ population was much smaller, but these cells also showed dynamic changes during ovulation. An increased number of M2 Mφs was observed after 48 h of HCG treatment, while the number of M1 Mφs decreased dramatically. This finding suggests that during corpus luteum formation, the microenvironment around ovulatory follicles changes from an inflammatory to an anti‐inflammatory state. Coculture of newborn ovaries with M0, M1 and M2 Mφs further supported the stimulatory effects of M1 Mφs on primordial follicles. However, no obvious effect was found in the M0 group, and to our surprise, we observed inhibitory effects of M2 Mφs on primordial follicles. Therefore, all the results suggest that during ovulation, alterations in the local microenvironment mediated by activated M1/M2 subtypes regulate the selective activation of primordial follicles.

Within the ovary, Mφs are involved in various aspects of ovarian function via their diverse activities, including phagocytosis in atresia and luteolysis, matrix dissolution and tissue remodelling in ovulation and corpus luteum formation, and the secretion of growth factors and cytokines during follicular growth.^21^, ^43^ Although direct interactions between Mφs and primordial follicles have not been observed, there is the possibility that Mφs function in a paracrine manner. Many Mφ‐derived cytokines and growth factors, including epidermal growth factor, insulin‐like growth factor, vascular endothelial growth factor, and TGF a and b, have been shown to participate in the regulation of primordial follicles.^44^, ^45^, ^46^, ^47^, ^48^ However, because these growth factors are also produced by other ovarian cells, such as granulosa cells and thecal cells, the role of Mφs in follicular growth is often neglected. Recently, Mφ‐derived EVs were reported to modulate inflammatory processes. EVs are nanosized vesicles with a lipid bilayer that are secreted by cells for intercellular communication. These vesicles are characterised by their parent cells and carry cell‐specific proteins, lipids or RNAs to affect the physiological or pathological state of target cells. EVs derived from different Mφs (M0, M1 and M2) contain different bioinformation, mainly miRNAs or lncRNAs, that exert various functions in tissue homeostasis and the progression of various diseases, such as cancer, heart disease and atherosclerosis.^49^ The PI3K/mTOR signalling pathway plays a key role in follicular activation.^5^ By monitoring the phosphorylation of PI3K/mTOR signalling downstream effectors, our study demonstrated the differential regulation of this signalling pathway by M0, M1 and M2 Mφs and their derived EVs. In addition, we found that two specific miRNAs, miR‐107 and miR‐99a‐5p, from M1‐ and M2‐EVs, by targeting PTEN and mTOR respectively, participated in modulating this signalling pathway and follicular activation in an opposing manner. A recent study revealed that EVs derived from tissue‐infiltrating Mφs that carried an ERCC1‐XPF DNA repair defect triggered pro‐inflammatory reactions in target cells via mTOR activation.^50^ EVs derived from M2 Mφs were also reported to have a therapeutic effect in a rat knee osteoarthritic model or in glioma through inhibition of the PI3K/mTOR signalling pathway.^51^, ^52^ Consistent with the results of these studies, our results further demonstrated the differential regulation of primordial follicle activation by polarised Mφs through their derived EVs and the shuttling of specific miRNAs.

Recently, an increasing number of studies have revealed that ovarian ageing is associated with a pro‐inflammatory environment. Distinct from injury‐ or pathogen‐induced acute inflammation, noninfectious chronic low‐grade inflammation, also called inflamm‐ageing, is not only a hallmark of the normal ageing process, but also associated with various age‐related pathologies. In accordance with previous studies, our result demonstrated the increased Mφs, especially M1 Mφs in the aged ovary, together with differential expression and distribution of M1 and M2 Mφs and pro‐ or anti‐inflammatory factors between young and aged ovaries.^23^, ^53^ Such pro‐inflammatory environment in aged ovaries means that anti‐inflammatory therapy may be an option to reverse the side effects of chronic inflammation on ovarian function. In fact, experiments in which M2 Mφ‐derived EVs were intravenous injected into aged mice revealed the recovery of ovarian functions with increased fertility and the improvement of oocyte quality. Furthermore, we observed a transition of ovarian inflammation accompanied by decreased expression of pro‐inflammatory cytokines and reduced numbers of M1 Mφs. On the contrary, in M1‐EVs‐treated mice, we found a greater deterioration of the ovarian microenvironment, with decreased oocyte quality and fertility. Meanwhile, we detected intensified fibrosis in M1‐EVs‐treated but not in M2‐EVs‐treated ovaries. The role of M2 Mφs to deposit collagen in the ECM, which promotes fibrosis has been reported in the pathogenesis of many end‐stage chronic inflammatory diseases, such as liver cirrhosis and idiopathic pulmonary fibrosis.^54^ Weather M2 Mφs participate in the ovarian fibrosis remains controversial.^22^ In the study, M2‐EVs treatment could not induce ovarian fibrosis in aged ovaries. Moreover, treatment with M0‐EVs contained agomir‐99a‐5p on stromal cells isolated from old mice demonstrated the inhibition of mTOR signalling pathway with downregulated expression of inflammatory factors. These results suggest the anti‐inflammatory effects of M2‐EVs on aged ovaries. One recent study reported that conditional medium from polarised M2 Mφs did not induce profibrotic responses in lung fibroblasts or alveolar epithelial cells in vitro.^55^ To be noted, inflammation also plays a fundamental role in fibrosis. For example, the major pro‐inflammatory cytokines IL‐1β, IL‐6 and TNF‐α have profibrotic functions, while the anti‐inflammatory cytokine IL‐10 is antifibrotic.^56^, ^57^ Therefore, our study illuminates the stimulatory effects of pro‐inflammatory M1‐EVs on ovarian fibrosis, whereas the anti‐inflammatory effects of M2‐EVs are required for the amelioration of ovarian microenvironment and the resulted improvement of ovarian function in old mice. M2‐EVs is a good candidate to delay ovarian ageing.

In summary, we introduce a new concept about selective primordial follicle activation induced by cyclic Mφ fluctuations during ovulation. Different subtypes of Mφs, M1 and M2 Mφs, exert differential regulation on primordial follicles through derived EVs and specific miRNAs. The increase in pro‐inflammatory M1 Mφs contributes to the inflammatory microenvironment and the accelerated depletion of ovarian reserve during the ageing process. Finally, the ageing‐related decline of ovarian function could be effectively rescued by treatment with M2‐EVs (Figure 7G). Future study is still required to delve into the mechanism about how M2‐EVs and their contents reverse ovarian stromal cell function and improve follicular development in aged ovaries. Since chronic inflammation also exists in other ovarian pathologies, such as PCOS and POF,^58^, ^59^ we propose M2‐EVs may represent a new therapeutic approach for resolving chronic inflammation‐induced ovarian pathologies.

## 4 MATERIALS AND METHODS

### 4.1 Mice

Mice were obtained from Nanjing Medical University and housed in Animal Core Facility of Nanjing Medical University (Nanjing, China). Mice were maintained under a 12 h dark/12 h light cycle at 22°C with free access to food and water. The experimental protocol was approved by the Committee on the Ethics of Animal Experiments of Nanjing Medical University. Female ICR mice at 8 weeks of age were used for natural‐ and superovulation to collect ovaries. Ovaries with natural ovulation were collected from mice at estrus stage and ovulation was manifested by detecting oocytes in the ampullary segment of the oviduct. Superovulation was performed with one intraperitoneal injection of PMSG (5 IU, Ningbo, China) and followed by another injection of HCG (5 IU, Ningbo, China) 48 h later. Ovaries were harvested at different timepoints (PMSG 48 h and HCG 10–48 h) for further analysis. Mice at 10‐month of age were used for intravenous injection of Mφ‐EVs and the mating experiment was done with adult male mice of the same strain (8 weeks old).

### 4.2 Rapamycin treatment and in vitro fertilisation

PMSG‐primed mice were received one injection of mTOR inhibitor, rapamycin (2 mg/kg, Sigma) 1 h before HCG treatment and ovaries and oocytes were collected, respectively, 14–16 h later. Collected ovaries were used to evaluate the effect of rapamycin on primordial follicle activation and oocytes were used to check its effect on oocyte maturation, fertilisation and early embryonic development. For IVF studies, donor sperm were first collected from male mice into G‐IVF media (Vitrolife) and incubated under oil for 1 h at 37°C in 5% CO_2_ for capacitation. MII oocytes were then placed into 250 μl of media with sperm (2–3 × 10^5^/ml) for fertilisation. Six hours later, zygotes with clear pronuclei were transferred into fresh G‐1 media (Vitrolife, Sweden) overnight until the two‐cell embryonic stage.

### 4.3 Stromal cells isolation and culture

Ovaries were collected and transferred into αMEM (Gibco, USA) containing 0.1% collagenase IV (Sigma, USA). To obtain stromal cells, corpora lutea were removed using 27 G needle. Then, all samples were incubated at 37°C for 30 min with gentle shaking every 5 min. Stromal cells were purified using 40‐μm filter (Millipore, USA) and the medium containing stromal cells were centrifugation at 1000 *g* for 5 min. Stromal cells were cultured in αMEM with 10% foetal bovine serum (FBS, Gibco), penicillin/streptomycin (100 μg/ml). After cultured for 30 min, removed un‐adhered cells and changed the culture medium.

### 4.4 BMDM isolation and polarisation

Bone marrow was flushed from femur and tibia bones using 10 ml syringe with 21 G needles and cells were cultured in RPMI 1640 medium (Bio‐sharp, China) with 10% FBS (10099141, Gibco), penicillin/streptomycin (100 μg/ml) and 10% (vol/vol) conditional medium of L929 mouse fibroblasts for 7 days.^60^, ^61^ For M1 Mφs activation, cells were incubated for 48 h with 100 ng/ml LPS (Solarbio, China) and for M2 Mφs activation, cells were incubated for 48 h with 20 ng/ml IL‐4 (Peprotech, USA). The efficiency of polarisation was checked by flow cytometry analysis with 98.9% in M1 induction and 98.2% in M2 induction, respectively. For L929 conditional medium collection, L929 cells were cultured in RPMI 1640 containing with 10% FBS and 1% penicillin/streptomycin. Cells should become confluent in 2–3 days and supernatants were collected 2 days later.

### 4.5 Isolation of EVs derived from BMDM

BMDM, including M0, M1 and M2 Mφs, were starved for 2 days under serum‐free conditions. Supernatants were collected and centrifuged at 300 *g* for 10 min at 4°C to remove cells and then centrifuged at 2000 *g* for 10 min to remove dead cells. After centrifuging at 10 000 *g* for 30 min to remove cell debris, the supernatants were ultracentrifuged (L‐100XP, Beckman Coulter, Germany) at 150 000 *g* for total 150 min by using a SW32Ti rotor to isolate EVs. The pellet was then washed with PBS to remove contaminating proteins and centrifuged again at 150 000 *g* for another 90 min. The produced EVs were diluted in 150 ml of PBS. Particle size and concentration were measured by NTA (Particle Metrix, Germany).

### 4.6 EVs miRNA treatment

Levels of target miRNA can be enhanced using molecular mimic ‘agomir’. Agomir‐107 and agomir‐99a‐5p (RiboBio, China) were transfected into M0‐EVs by lipofectamine 2000 (Invitrogen, USA) according to the instructions of the manufacturer. Briefly, the EVs pellet isolated from 200 ml of conditional media were resuspended with 100 μl of PBS containing 100 nM agomir plus 2 μl of Lipofectamine 2000 and incubated at 37°C for 12 h. After washing with PBS, agomir‐containing EVs were pelleted by ultracentrifugation at 120 000 *g* for 2 h. Finally, 150 μl of PBS was add to resuspend the pellet, and same concentration of M0‐EVs and M0‐agomirs were used for ovarian treatment. For cells and ovarian tissue culture, stromal cells or ovaries were incubated with M0e‐agomirs at 37°C for 24 h.

### 4.7 In vitro culture of newborn ovaries and kidney capsule transplantation

Ovaries were collected from P2.5 female pups and cultured on Millicell inserts (Millipore) in 24‐well culture plates (NEST, China) containing 400 μl of ovarian culture medium. The plates were placed in 37°C tissue culture incubator with 5.0% carbon dioxide. Ovarian culture medium was αMEM (Gibco) supplemented with 0.23 mM pyruvic acid, 50 mg/L streptomycin sulphate (Biofil), 75 mg/L penicillin (Biofil, China), 0.03 U/ml follicle stimulating hormone (Ningbo, China) and 3 mg/ml bovine serum albumin (Sigma, USA). For in vitro coculture experiment with M0, M1 and M2 Mφs, BMDMs (2 × 10^5^) were first plated in 12‐well culture plates for polarisation 48 h, respectively, and then cocultured with ovaries for 24 h. After coculture treatment, some ovaries in each group were then transferred and cultured into control medium for 72 h to evaluate ovarian development. Ovaries were treated with M0‐, M1‐ and M2‐EVs or M0‐EVs containing agomir at 100 nM for 24 h. After treatment with agomir‐containing M0‐EVs, paired ovaries treated with or without agomir‐containing M0‐EVs were transplanted under the kidney capsules of bilaterally ovariectomised adult females. After 2 weeks of transplantation, mice were sacrificed to detect follicular development.

### 4.8 Intravenous injection and fertility test

Female mice at the age of 10 months old were grouped and received a total volume of 100 μl solution containing 3.6 × 10^9^ particles of M1 or M2 Mφ‐derived EVs or the same volume PBS via tail intravenous injection every 5 days. After five times of injection, ovaries were collected to evaluate follicular development. Some mice were for superovulation and MII oocytes were collected for morphology and JC‐staining to evaluate oocyte quality. To perform fertility test experiments, M1/M2‐EVs or PBS‐treated mice were mated with the same strain of fertile males (1:1). The pregnancy rate and first litter size were then analysed.

### 4.9 Flow cytometry

Isolation and separation of the ovary and subsequent flow cytometry were performed as previously described.^62^ For flow cytometry analysis, Mφs were fixed with 4% paraformaldehyde and permeabilised with PBS contained 0.25% Triton X‐100 (PBST). Then, primary antibodies labelled with fluorescence were added and incubated for 1 h at 4°C. After washed two times with PBS, Mφs were analysed by flow cytometer (BDVerse, USA). Mφs in ovaries were defined as CD45^+^/F4/80^+^ cells, M1 Mφs were defined as CD45^+^ F4/80^+^ CD11c^+^ CD206^−^ cells and M2 Mφs were defined as CD45^+^ F4/80^+^ CD11c^−^ CD206^+^ cells. The antibodies used in this study are as follows: PE anti‐mouse CD45 (561087, BD, USA), PE‐Cy7 anti‐mouse F4/80 (123113, Biolegend, USA), FITC anti‐mouse CD11c (117305, Biolegend) and APC anti‐mouse CD206 (141707, Biolegend).

### 4.10 Immunohistochemistry and immunofluorescence

Samples were fixed in 10% formalin overnight and embedded in paraffin and sectioned to a thickness of 5 μm. For immunohistochemistry, sections were deparaffinised, rehydrated and endogenous peroxidase activity was blocked by incubating in 3% hydrogen peroxide in methanol for 15 min. The antigen of ovarian sections was retrieved by high temperature (95°C) for 16 min in sodium citrate buffer (pH 6.0). Then, the sections were blocked for 60 min at room temperature with blocking goat serum (ZSGB‐Bio, China) for 1 h and incubated with primary antibodies at 4°C overnight. The sections were coloured with diaminobenzidine reagent. For immunofluorescence, the sections were incubated with secondary antibody (Alexa Fluor 488, Alexa Fluor 594, Invitrogen) at 37°C for 1 h. The nuclei were then counterstained with Hoechst 33342 (Invitrogen) for 10 min. The antibodies were as follows: FOXO3a (CST #12829s, USA), PCNA (CST #27214), DDX4 (abcam #33864, USA), F4/80 (abcam#6640, USA), iNOS (Proteintech #22226, China), CD206 (Proteintech #18704), α‐tubulin (Siama #2168, USA) and TUNEL Assay Kit (YEASEN, China).

### 4.11 Follicle counting

In vitro‐cultured ovaries and superovulated ovaries were collected and fixed in 10% formalin overnight for serial sections (5 μm) and haematoxylin and eosin staining. To evaluate follicular development, all follicles were counted at every fifth section using the fractionator and nucleator principles. To evaluate the activation of primordial follicles, two serial sections from the largest cross‐section through the center of each ovary were chosen for FOXO3a staining, and those primordial follicles with plasma translocate staining were counted as activated follicles. All sections were counted by two independent individuals for comparison.

### 4.12 Immunoblotting analysis

Ovaries were lysed in RAPI (P0013B, Beyotime Institute of Biotechnology, China) with 1% protease inhibitor (MCE, USA). Proteins were separated by electrophoresis by 10% sodium dodecyl sulphate‐polyacrylamide gel electrophoresis and transferred into polyvinylidene fluoride membranes (Bio‐Rad, USA). The membranes were blocked with 5% nonfat‐dry milk for 60 min and incubated at 4°C overnight with following primary antibodies: p‐RPS6 (9234, CST, USA), RPS6 (2217, CST, USA), p‐AKT (9271, CST, USA), AKT (9272, CST, USA), β‐tubulin (Abbkine, China), β‐actin (Abbkine), GAPDH (Abbkine), iNOS (60291, Proteintech) and CD206 (18704, Proteintech). The membranes were washed with TBST 10 min for three times and incubated with secondary antibodies for 60 min. The signals were enhanced through enhanced chemiluminescence (34094, Thermo Fisher Scientific, USA).

### 4.13 Quantitative real‐time PCR

Total RNAs were isolated from ovaries or Mφs by TRIzol reagent (Invitrogen). Then, 500–1000 ng RNA/per reaction were reverse transcribed into cDNAs using FastQuant RT Kit (TIANGEN Biotech, China). Quantitative real‐time PCR was then performed using SYBR Green Mix (Applied Biological Materials, Canada) in an ABI StepOnePlus platform (Thermo Fisher Scientific). The primer sequences are listed in Table S1. The specificity of PCR products was assessed by melting curve analyses and amplicon size was determined by electrophoresis in 2% agarose gels.

### 4.14 Transmission electron microscopy

EVs pellet obtained were fixed in 100 μl of 2.5% glutaraldehyde overnight at 4°C. Then, EVs were processed and wrapped in epoxypropane resin following standard TEM procedures. EVs were observed by TEM (Tecnai G2 Spirit Biotwin, FEI, USA).

### 4.15 ∆Ψm measurement

Briefly, the collected MII oocytes were washed with M2 medium several times and then transferred into MII medium containing staining solution of JC‐1 ∆Ψm assay kit (YEASEN) and incubated in the dark for 30 min. Then, oocytes were washed with M2 medium to remove the excess staining solution. All the live oocytes were observed and relative fluorescence intensity was calculated by confocal microscopy.

### 4.16 Statistical analysis

GraphPad Prism 7.0 and Excel 2016 were used to performed to the values are presented as mean ± standard deviation. One‐way analysis of variance analysis was used to determine significant differences between treatment and control groups. All experiments were repeated at least three times, and a value of *p* < .05 was statistically significant.

## CONFLICT OF INTEREST

The authors declare they have no conflicts of interest.

## Supporting information

Supporting information

Supporting information

Supporting information
