# Supplementary figures and images for: Macrophage‐derived extracellular vesicles regulate follicular activation and improve ovarian function in old mice by modulating local environment
Source: Clin Transl Med. 2022 Oct 13;12(10):e1071. doi: 10.1002/ctm2.1071 (PMC9561167; doi:10.1002/ctm2.1071)

Fig S1


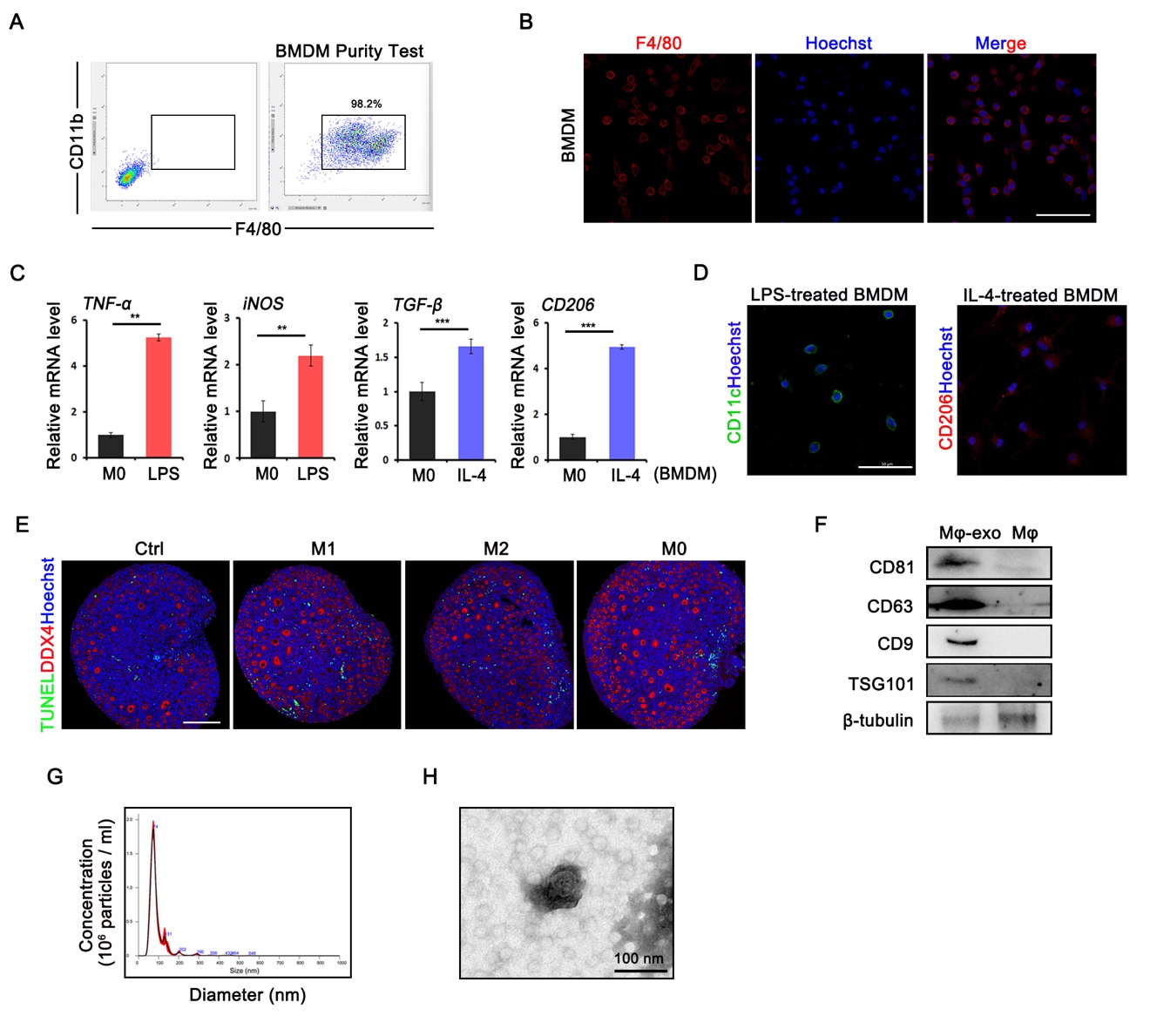


Fig S2


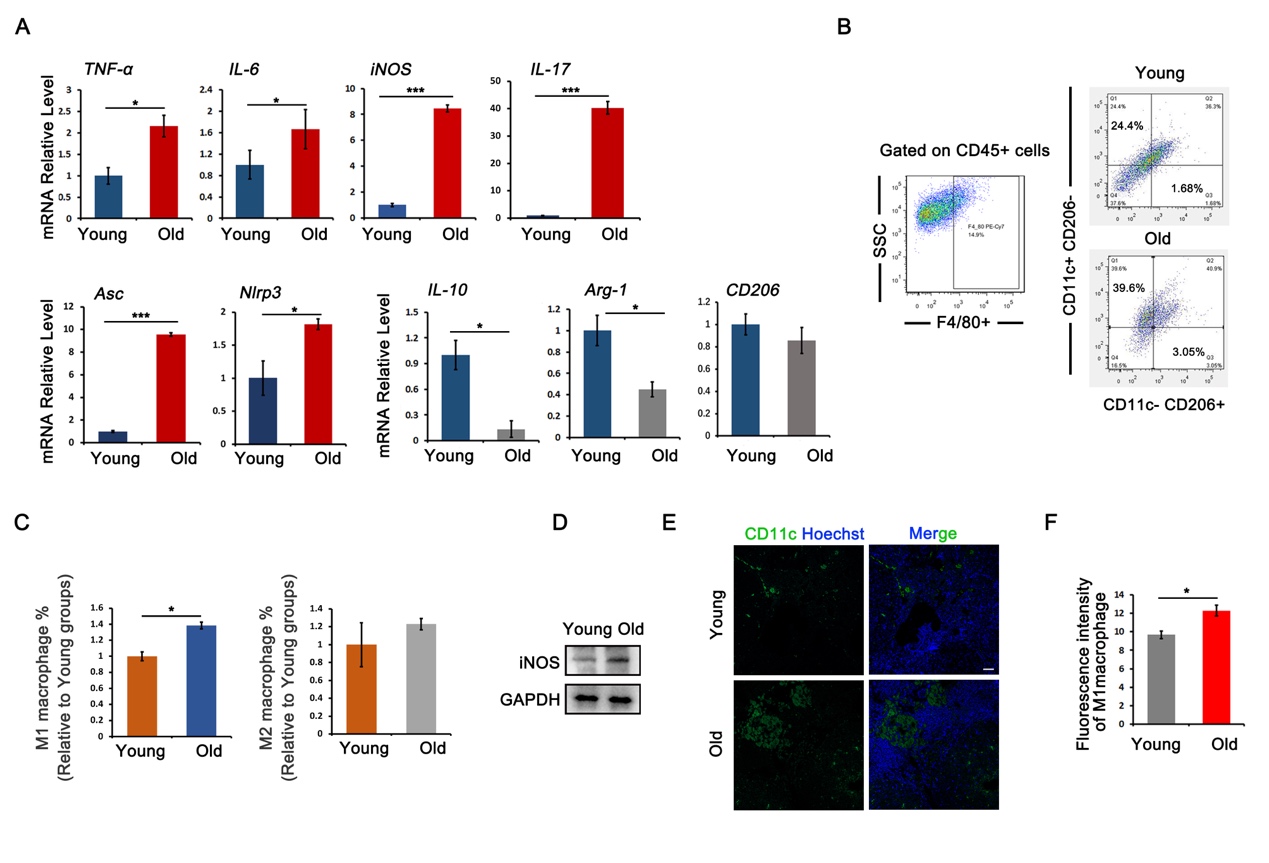


Fig S3


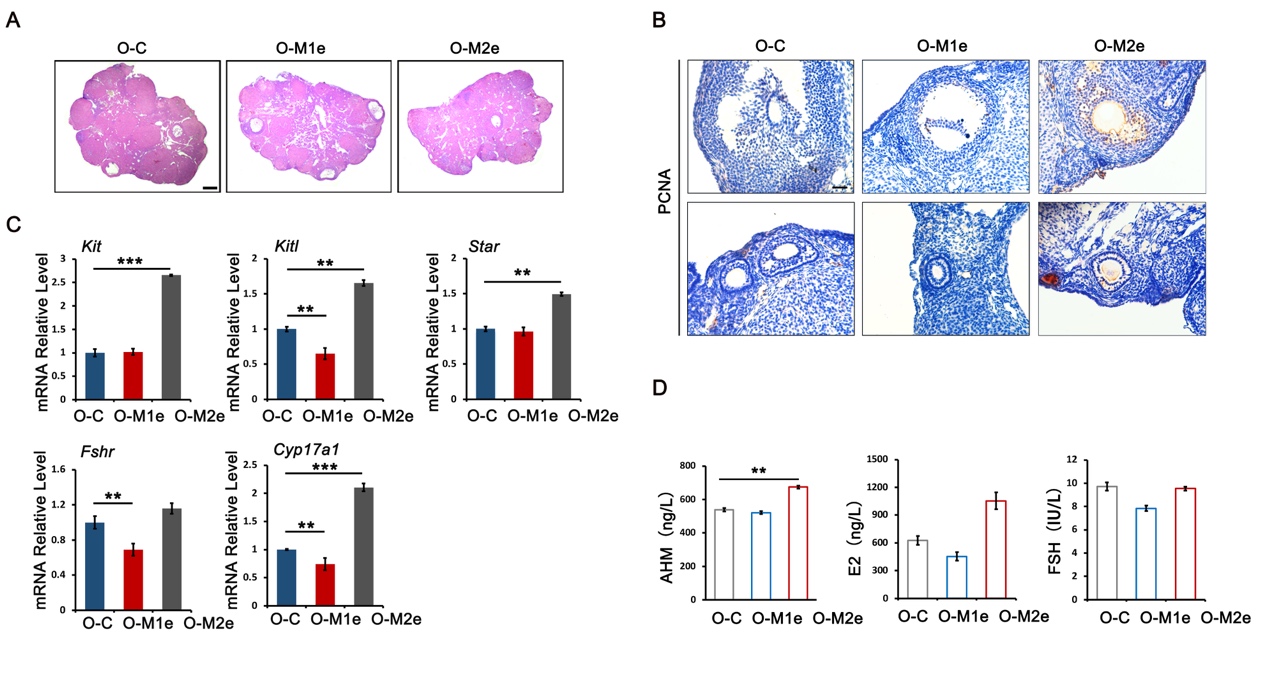


Fig S4


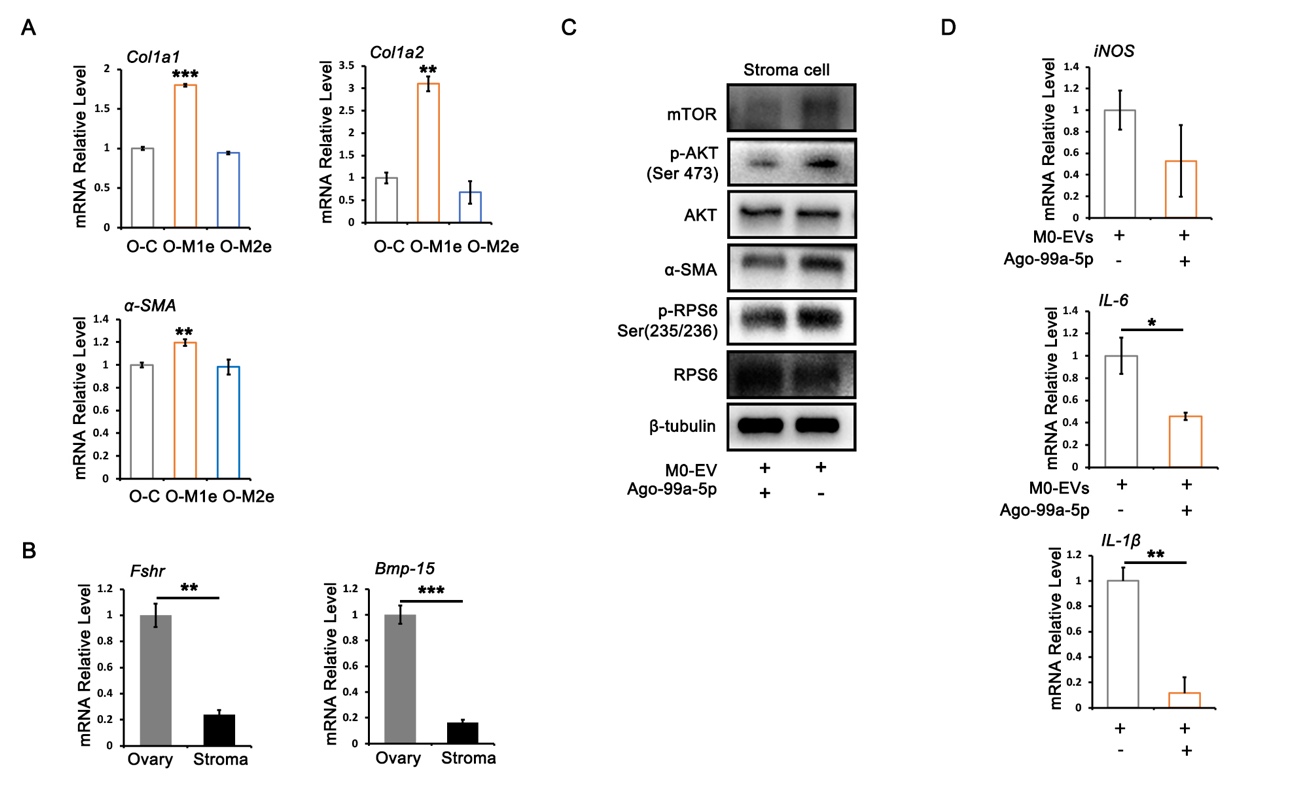

Supplement: Supplementary file 1 — Supporting information [file CTM2-12-e1071-s003.docx]
