# Supplementary material for: Macrophage‐derived extracellular vesicles regulate follicular activation and improve ovarian function in old mice by modulating local environment
Source: Clin Transl Med. 2022 Oct 13;12(10):e1071. doi: 10.1002/ctm2.1071 (PMC9561167; doi:10.1002/ctm2.1071)
Supplement: Supplementary file 2 — Supporting information [file CTM2-12-e1071-s001.docx]

**Supplementary Figure Legend**

**Figure S1. BMDM acquisition and characterization of BMDM-derived extracellular Vesicles.**

(**A**) BMDMs purity test. BMDMs were defined as CD11b^+^ F4/80^+^ cells. (**B**) Immunofluorescence staining of F4/80 in BMDM. Red: F4/80, Blue: Hoechst (**C**) Relative expression of M1 Mφs marker genes (*TNF-α, iNOS*) and M2 Mφs marker genes (*TGF-β, CD206*) in LPS treated BMDM and IL-4 treated BMDM. (**D**) Immunofluorescence analysis of CD11c in LPS-treated Mφs and CD206 in IL4-treated Mφs. Green: CD11c, Red: CD206, Blue: Hoechst (**E**) TUNEL assay of follicular apoptosis in control ovaries and M1, M2 and M0 Mφs treated ovaries. Green: TUNEL signal, Red: DDX4, Blue: Hoechst. (**F**)Western blot analysis of EVs-related markers CD81, CD63, CD9 and TSG101 expression in Mφ-derived EVs. The expression of β-tubulin was used as internal control. (**G**) Nanoparticles tracking analysis of size distribution and concentration of Mφ-derived EVs. The M0-EVs particles had a typical modal size of 73.1-131.6 nm. The mean protein concentration and mean particle concentration of Mφ-derived EVs were 7.49 x 10^12^ particles/mL (**H**) Representative TEM image of Mφ-derived exosomes. Scale bars: 100 nm.

Data represent the mean ± SD of biological triplicate experiments. *, P < 0.05 and ***, P < 0.001, by one-way ANOVA analysis. Scale bars: 50 μm.

**Figure S2. Increased pro-inflammatory environment in aging ovary.**

Young (8 week) and old (12 month) ovaries were collected. (**A**) Relative expression of pro-inflammatory genes (*TNF-α, IL-6, iNOS, IL-17*), inflammasome-associated genes (*Asc and Nlrp3*) and anti-inflammatory genes (*IL-10, Arg-1, CD206*) in young and aged ovaries. (**B**) Flow cytometry analysis of M1 and M2 Mφs in young and aged ovaries. M1 Mφs were defined as CD45^+^ F4/80^+^ CD11c^+^ CD206^-^ cells, M2 Mφs were defined as CD45^+^ F4/80^+^ CD11c^-^ CD206^+^ cells. (**C**) The proportion of M1 and M2 Mφs in young and aged ovaries. (**D**) Western blot of iNOS expression in young and aged ovaries. The expression of GAPDH was used as internal control. (**E**) Immunofluorescent labeling of M1 Mφs in young and aged ovaries. (Green, CD11c; Blue: Hoechst 33342) (**F**) Fluorescent intensity of M1 Mφs in young and aged ovaries.

Data represent the mean ± SD of biological triplicate experiments. *, P < 0.05 and ***, P < 0.001, by one-way ANOVA analysis. Scale bars: 50 μm.

**Figure S3. M2-EVs improved ovarian function in aged ovaries.**

Mice at 10-month of age were tail intravenous injected with 100 μL PBS containing 15 μg M1-, M2-EVs (O-M1e and O-M2e) and same volume of PBS (O-C) 5 times for 28 days, respectively. (**A**) Histology of ovaries in O-C, O-M1e and O-M2e groups. (**B**) Immunostaining of PCNA in follicles in O-C, O-M1e and O-M2e groups. (**C**) The expression of follicle growth and development related genes *Kit*, *Kitl*, *Star*, *Fshr* and *Cyp17a1* in each group. (**D**) Levels of AMH, E2 and FSH in each group.

Data represent the mean ± SD of biological triplicate experiments. **, P < 0.01 and ***, P < 0.001, by one-way ANOVA analysis. Scale bars: 50 μm.

**Figure S4. M2-EVs changed inflammatory microenvironment in stromal cells in aged ovaries.**

Ovarian stromal cells were isolated from old mice (10 month of age) or mice treated with PBS (O-C), M1- or M2-EVs (O-M1e and O-M2e). (**A**) Relative expression of collagen-encoding genes *Col1a1*, *Col1a2* and *α-SMA* in stromal cells of O-C, O-M1e and O-M2e groups. (**B**) Relative expression of granulosa cell-related gene *Fshr* and oocyte development-related gene *Bmp-15* in isolated stromal cells and ovaries of old mice. (**C**) The expression of mTOR, p-AKT (Ser473) and p-RPS6 (Ser235/236) in stromal cells after treatment with M0-EVs or M0-EVs contained agomir-99a-5p (M0-EVs+Ago-99a-5p) for 24 h. The expression of AKT, RPS6 and β-tubulin were used as internal control. (**D**) The expression of pro-inflammatory genes in M0-EVs or M0-EVs+Ago-99a-5p treated stromal cells.

Data represent the mean ± SD of biological triplicate experiments. *, P < 0.05, **, P< 0.01 and ***, P < 0.001, by one-way ANOVA analysis.
