# Supplementary material for: Macrophage‐derived extracellular vesicles regulate follicular activation and improve ovarian function in old mice by modulating local environment
Source: Clin Transl Med. 2022 Oct 13;12(10):e1071. doi: 10.1002/ctm2.1071 (PMC9561167; doi:10.1002/ctm2.1071)
Supplement: Supplementary file 3 — Supporting information [file CTM2-12-e1071-s002.docx]

**Supplementary Table S1 Real-Time PCR Primers**

| *Gene* | Forward primer 5’-3’ | Reverse primer 5’-3’ |
| --- | --- | --- |
| *Actin* | CCGTAAAGACCTCTATGCC | CTCAGTAACAGTCCGCCTA |
| *Gdf9* | TCTTAGTAGCCTTAGCTCTCAGG | TGTCAGTCCCATCTACAGGCA |
| *Bmp15* | TCCTTGCTGACGACCCTACAT | TACCTCAGGGGATAGCCTTGG |
| *Amhr* | GCAGCACAAGTATCCCCAAAC | GTCTCGGCATCCTTGCATCTC |
| *Kit* | CTCCCCCAACAGTGTATTCAC | TAGCCCGAAATCGCAAATCTT |
| *Kitl* | GAATCTCCGAAGAGGCCAGAA | GCTGCAACAGGGGGTAACAT |
| *Star* | ATGTTCCTCGCTACGTTCAAG′ | CCCAGTGCTCTCCAGTTGAG |
| *Cyp17a1* | GCCCAAGTCAAAGACACCTAAT | GGTCGGTAGCGTATTTGGAAG |
| *Lhr* | CGCCCGACTATCTCTCACCTA | GACAGATTGAGGAGGTTGTCAAA |
| *Tnf-α* | GAGGCCAAGCCCTGGTATG | CGGGCCGATTGATCTCAGC |
| *iNOS* | TTCAGTATCACAACCTCAGCAAG | TGGACCTGCAAGTTAAAATCCC |
| *Il-1β* | ATGATGGCTTATTACAGTGGCAA | GTCGGAGATTCGTAGCTGGA |
| *Il-6* | ACTCAC CTC TTC AGA ACG AAT TG | CCATCTTTGGAAGGTTCAGGT TG |
| *Arg-1* | CCAGAAGAATGGAAGAGTCAGTGT | GCAGATATGCAGGGAGTCACC |
| *Il-10* | GACTTTAAGGGTTACCTGGGT TG | TCACATGCGCCTTGATGTCTG |
| *Tgf-β* | CTAATGGTGGAAACCCACAAC G | TATCGCCAGGAATTGTTGCTG |
| *Asc* | GACAGTACCAGGCAGTTCGT | AGTCCTTGCAGGTCAGGTTC |
| *Nlrp3* | CAGAGCCTACAGTTGGGTGA | TAGCAGTGAAGAGCAGTGCG |
| *Il-17* | GTGTCTCTGAGCTGTTG | AACGGTGAGGTAGTCTTG |
| *Gapdh* | GCGGGCGCTGGAGGAA | GGATCTTCATGAGGTAGTCA |
